# Supplementary material for: TIR domains of TLR family-from the cell culture to the protein sample for structural studies
Source: PLoS One. 2024 Jul 5;19(7):e0304997. doi: 10.1371/journal.pone.0304997 (PMC11226090; doi:10.1371/journal.pone.0304997)
Supplement: S1 File — Contains Figs (S1-S12) and Tables (S1-S25). (PDF) [file pone.0304997.s001.pdf]

Supplementary materials

TIR domains of TLR family - from the cell culture to the protein sample for structural studies

Lushpa V.A.[1,‡], Goncharuk M.V.[1,‡], Talyzina I.A.[1,#], Arseniev A.S.[1], Bocharov E.V.[1], Mineev K.S.[1,†,*], Goncharuk S.A.[1,*]

[1] Shemyakin-Ovchinnikov Institute of Bioorganic Chemistry of the Russian Academy of Sciences, Moscow 117997, Russia

[#] Current address: Department of Biochemistry and Molecular Biophysics, Columbia University, New York, NY, USA.

[†] Current address: Institute of organic chemistry and chemical biology, Goethe University Frankfurt, 60438 Frankfurt am Main, Germany

[‡] These authors contributed equally to this work

[*] Correspondence: konstantin.mineev@gmail.com (K.S.M.); ms.goncharuk@gmail.com (S.A.G.)

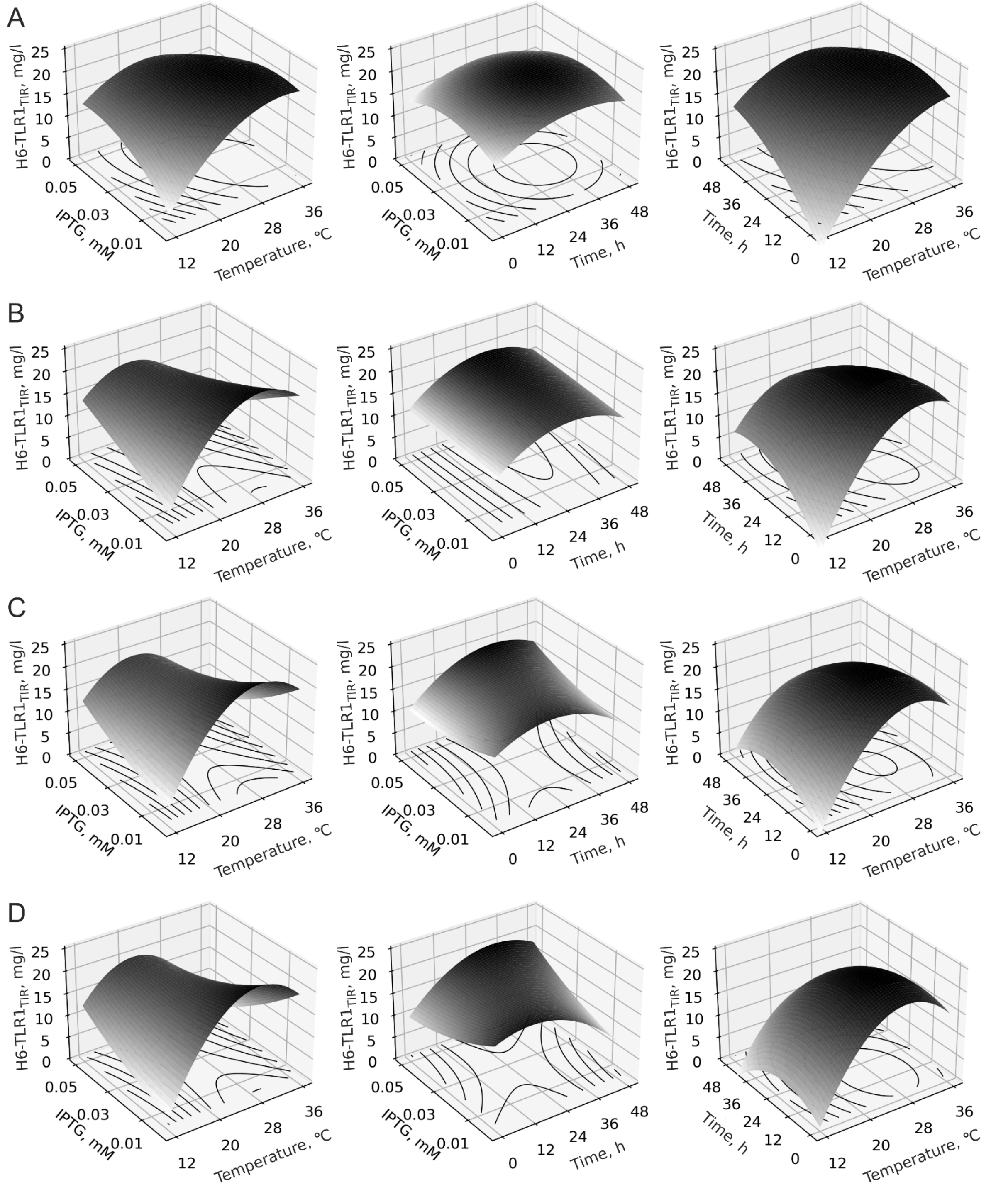

Figure S1. Influence of the number of points on the quality of the predicted model of H6-TLR1$_{TIR}$ expression. *Presented contour and surface response plots for H6-TLR1$_{TIR}$ production obtained based on the Box-Behnken design of experiments describe soluble protein yield as a function of different parameters of cellular growth. 150 (**A**), 34 (**B**), 27 (**C**) and 23 (**D**) experimental points were used. H6-TLR1$_{TIR}$ protein yield is shown in milligrams per liter of M9 minimal salts medium quantified based on target protein band intensities on the SDS-PAGE.*

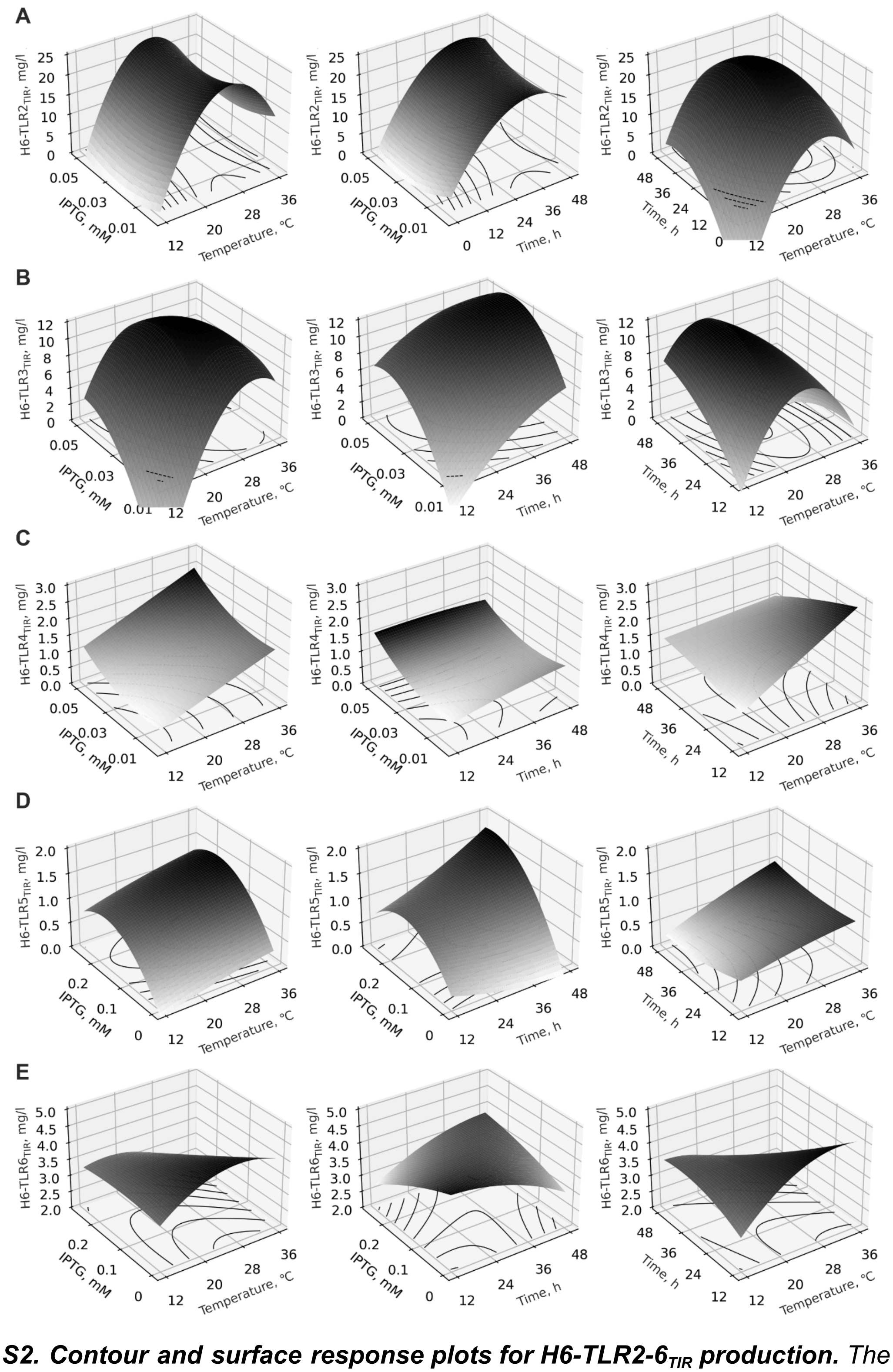

Figure S2. Contour and surface response plots for H6-TLR2-6$_{TIR}$ production. *The influence of IPTG concentration, post-induction temperature and time on soluble protein yield is shown for his-tagged TLR2$_{TIR}$* ***(A)****, TLR3$_{TIR}$* ***(B)****, TLR4$_{TIR}$* ***(C)****, TLR5$_{TIR}$* ***(D)*** *and TLR6$_{TIR}$. The H6-TLR$_{TIR}$ yield is in milligrams per liter of M9 minimal salts medium quantified based on target protein band intensities on the SDS-PAGE.*

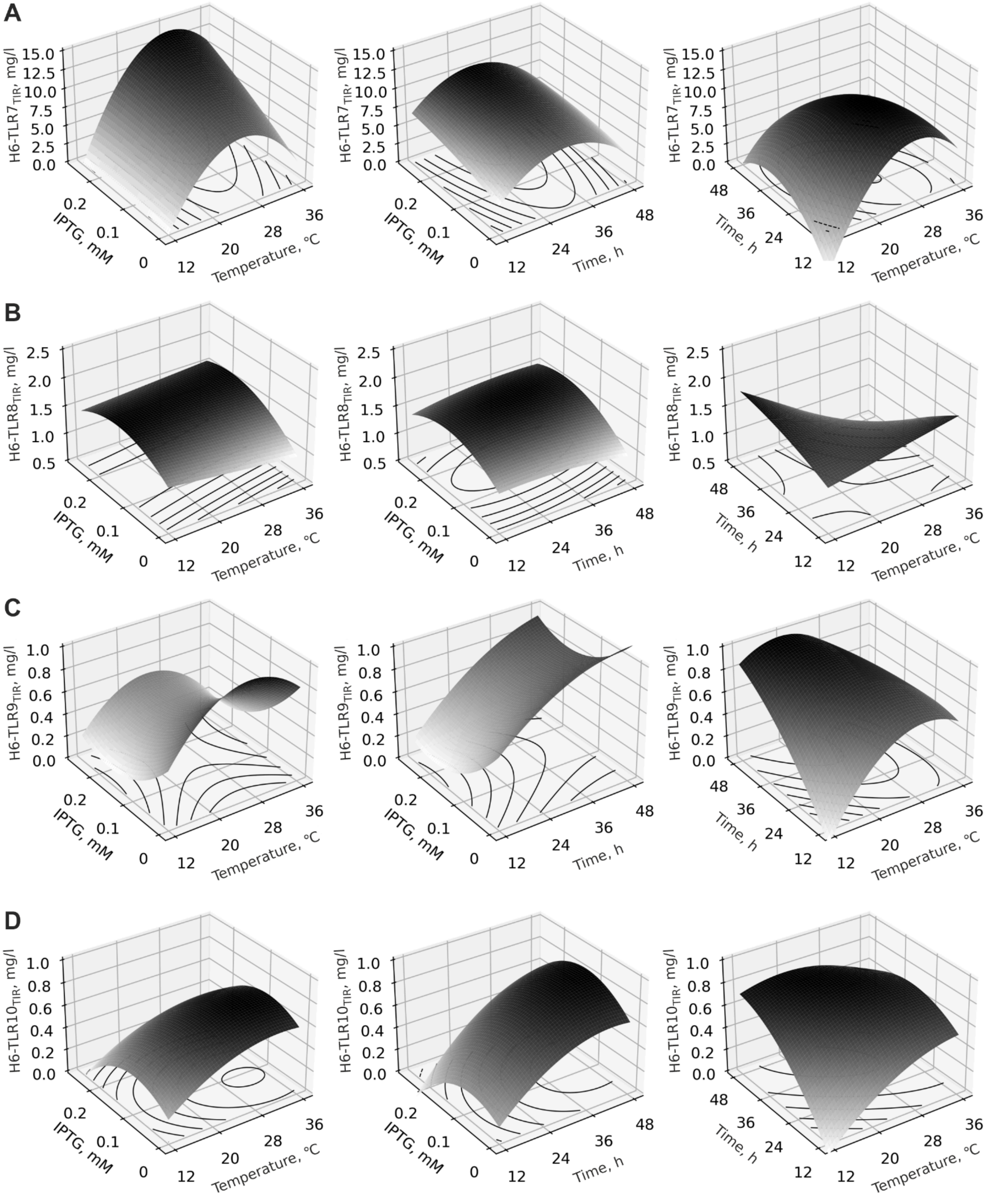

Figure S3. Contour and surface response plots for H6-TLR7-10$_{TIR}$ production. *The influence of post-induction temperature, post-induction time and IPTG concentration on soluble protein yield is shown for TLR7$_{TIR}$ (**A**), TLR8$_{TIR}$ (**B**), TLR9$_{TIR}$ (**C**) and TLR10$_{TIR}$ (**D**). The H6-TLR$_{TIR}$ yield is in milligrams per liter of M9 minimal salts medium quantified based on target protein band intensities on the SDS-PAGE.*

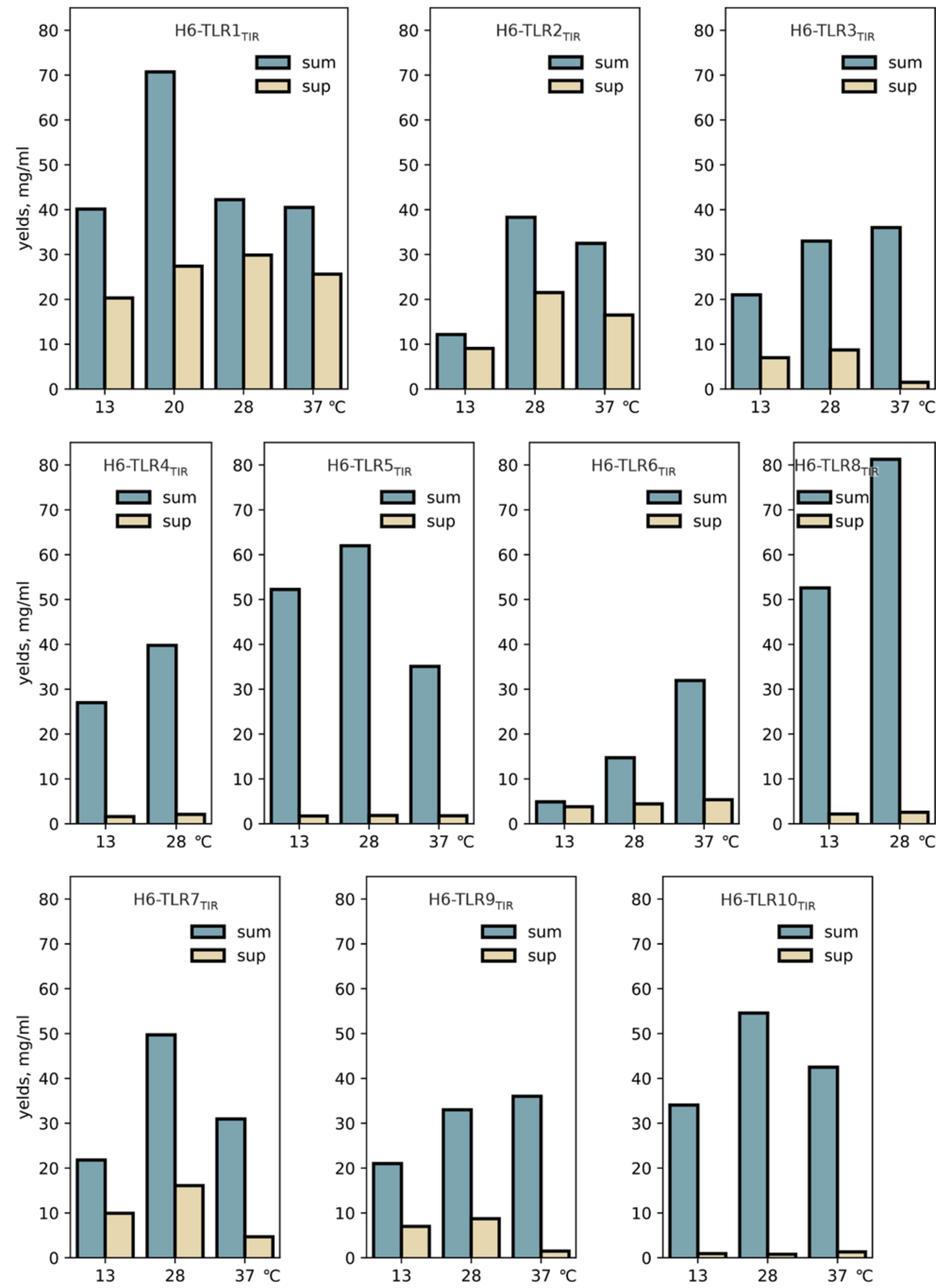

Figure S4. Screening the cultivation parameters for His-tagged TLR1-10$_{TIR}$. *The barplots illustrate the accumulation of total ("sum", in blue) and soluble ("sup", in beige) target protein under the set of parameters corresponding to maximal yield of total or soluble H6-TLR$_{TIR}$, correspondingly, for each of the analyzed temperatures (13, 28, 37 °C). H6-TLR$_{TIR}$ protein yields are shown in milligrams per liter of M9 minimal salts medium quantified based on target protein band intensities on the SDS-PAGE.*

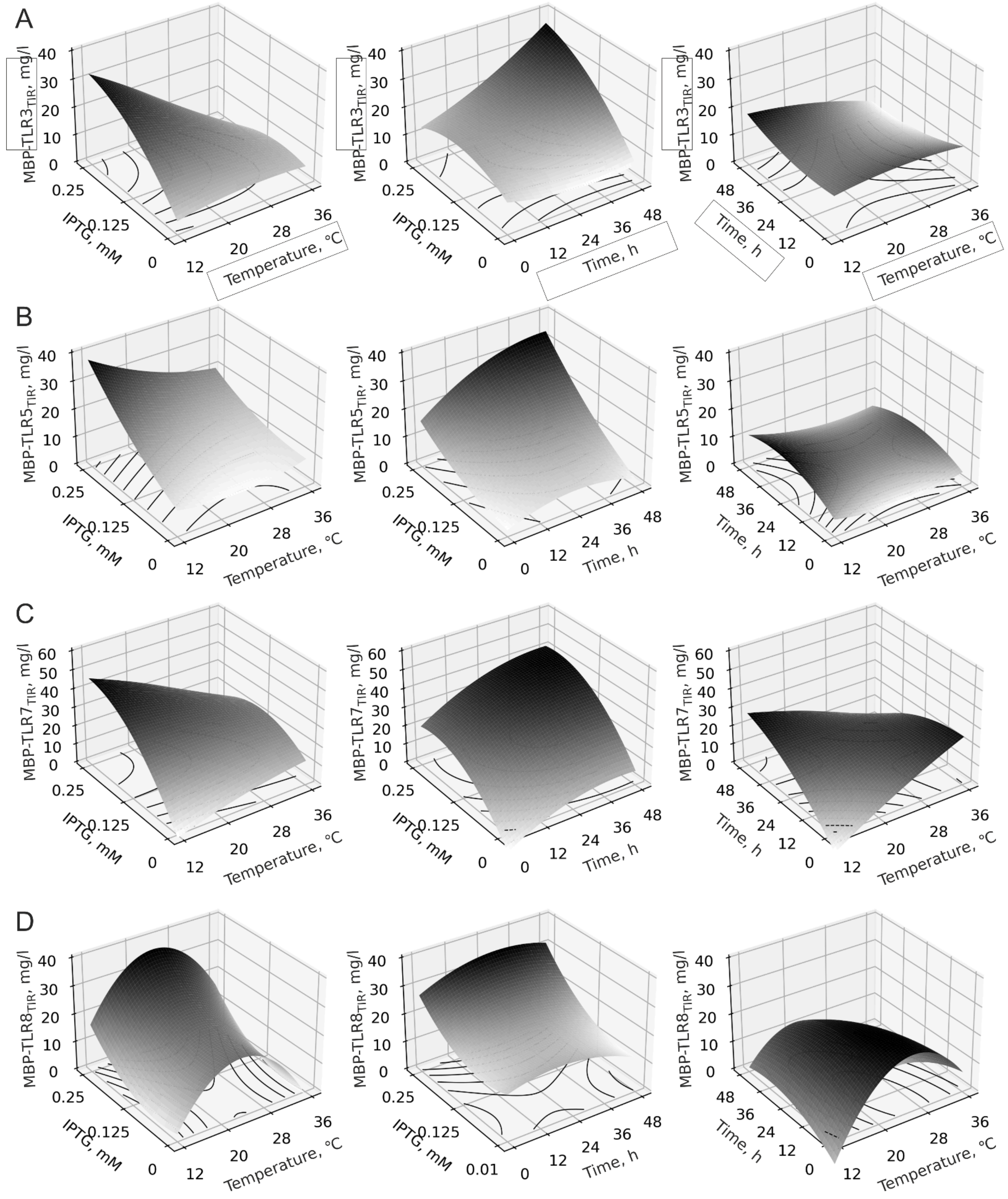

Figure S5. Contour and surface response plots for MBP-TLR3/5/7/8$_{TIR}$. *The influence of post-induction temperature, post-induction time and IPTG concentration on soluble protein yield is shown for MBP-TLR3$_{TIR}$ (**A**), MBP-TLR5$_{TIR}$ (**B**), MBP-TLR7$_{TIR}$ (**C**) and MBP-TLR8$_{TIR}$ (**D**). The MBP-TLR$_{TIR}$ yield is in milligrams per liter of M9 minimal salts medium quantified based on target protein band intensities on the SDS-PAGE.*

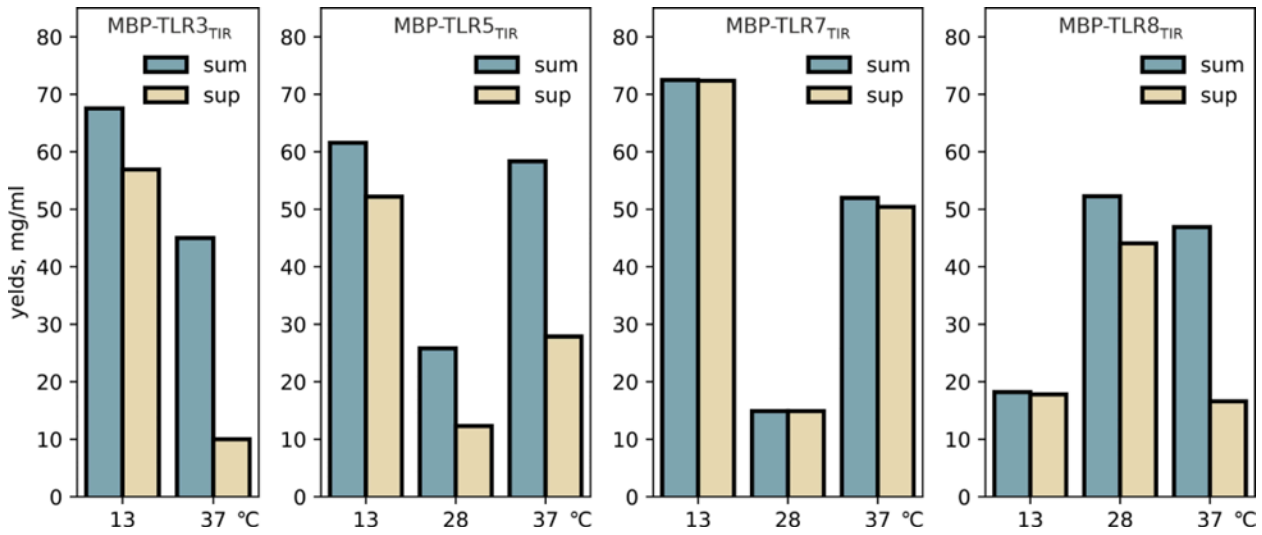

Figure S6. Screening the cultivation parameters for MBP-TLR$_{TIR}$. *The barplots illustrate the accumulation of total ("sum", in blue) and soluble ("sup", in beige) target protein under the set of parameters corresponding to maximal yield of total or soluble MBP-TLR$_{TIR}$, correspondingly, for each of the analyzed temperatures (13, 28, 37 °C). MBP-TLR3/5/7/8$_{TIR}$ protein yields are shown in milligrams per liter of M9 minimal salts medium quantified based on target protein band intensities on the SDS-PAGE.*

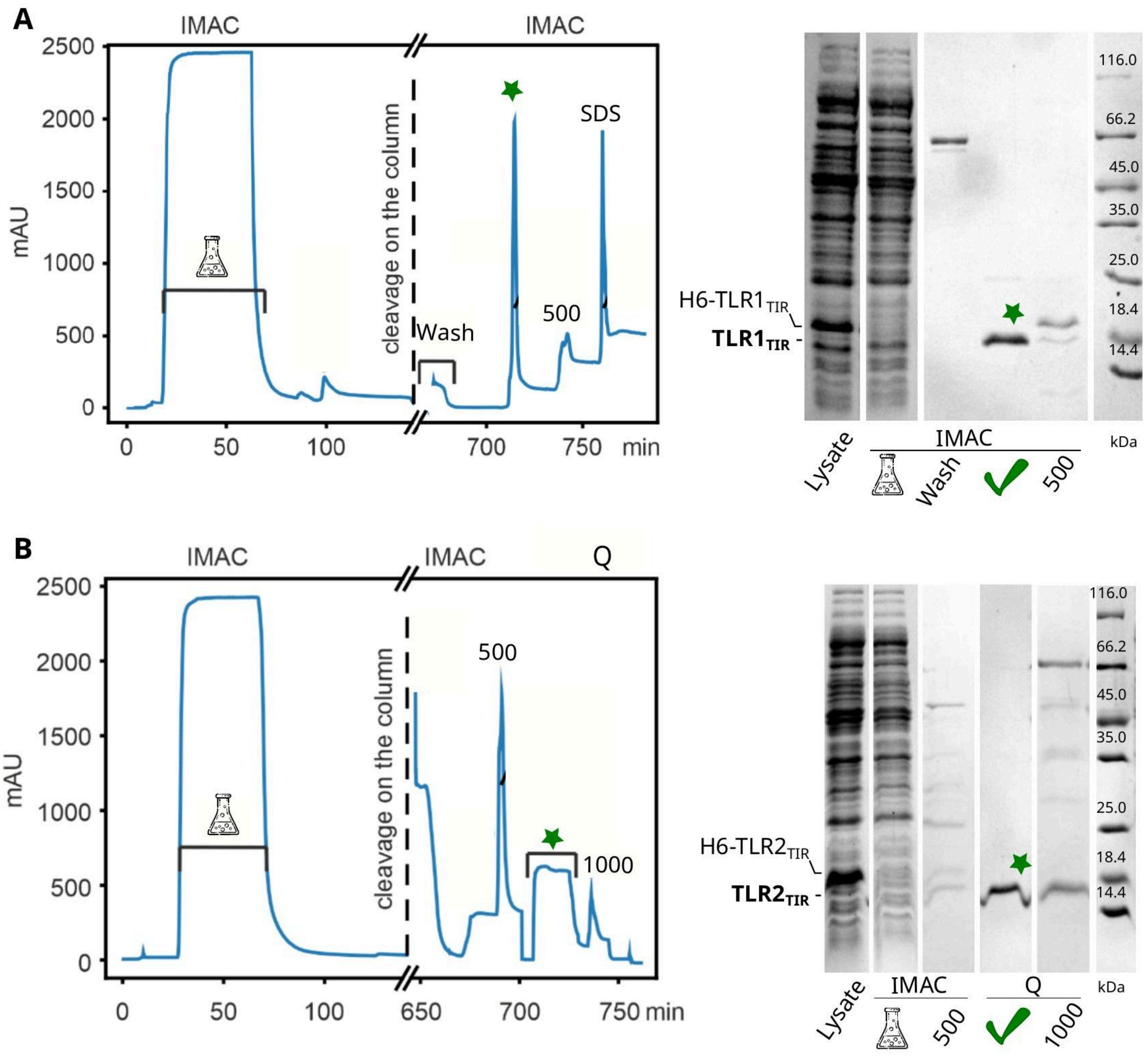

Figure S7. Purification summary for His-tagged TLR1$_{TIR}$ (A) and TLR2$_{TIR}$ (B). *From the left: combined chromatogram-views of the main purification steps (chromatography types used are indicated from the top). From the right: SDS-page lines corresponding to the main purification steps. Lines from different gels are separated with white spaces. "Lysate" - clarified lysate fraction applied to the column; picture of a flask - immobilized metal affinity chromatography (IMAC) flowthrough; "wash" – IMAC flowthrough after on-column digestion with thrombin; "500" – 500 mM imidazole IMAC eluate; "1000" – 1000 mM NaCl anion exchange chromatography (Q sepharose FF) eluate; green mark – fractions of purified TLR1/2$_{TIR}$; "SDS" - fraction of the protein precipitated on column during purification step. The green star denotes purified TLR1/2$_{TIR}$ band and corresponding peak at the chromatogram. Molecular weights marker is loaded from the right of the gel and protein bands are signed.*

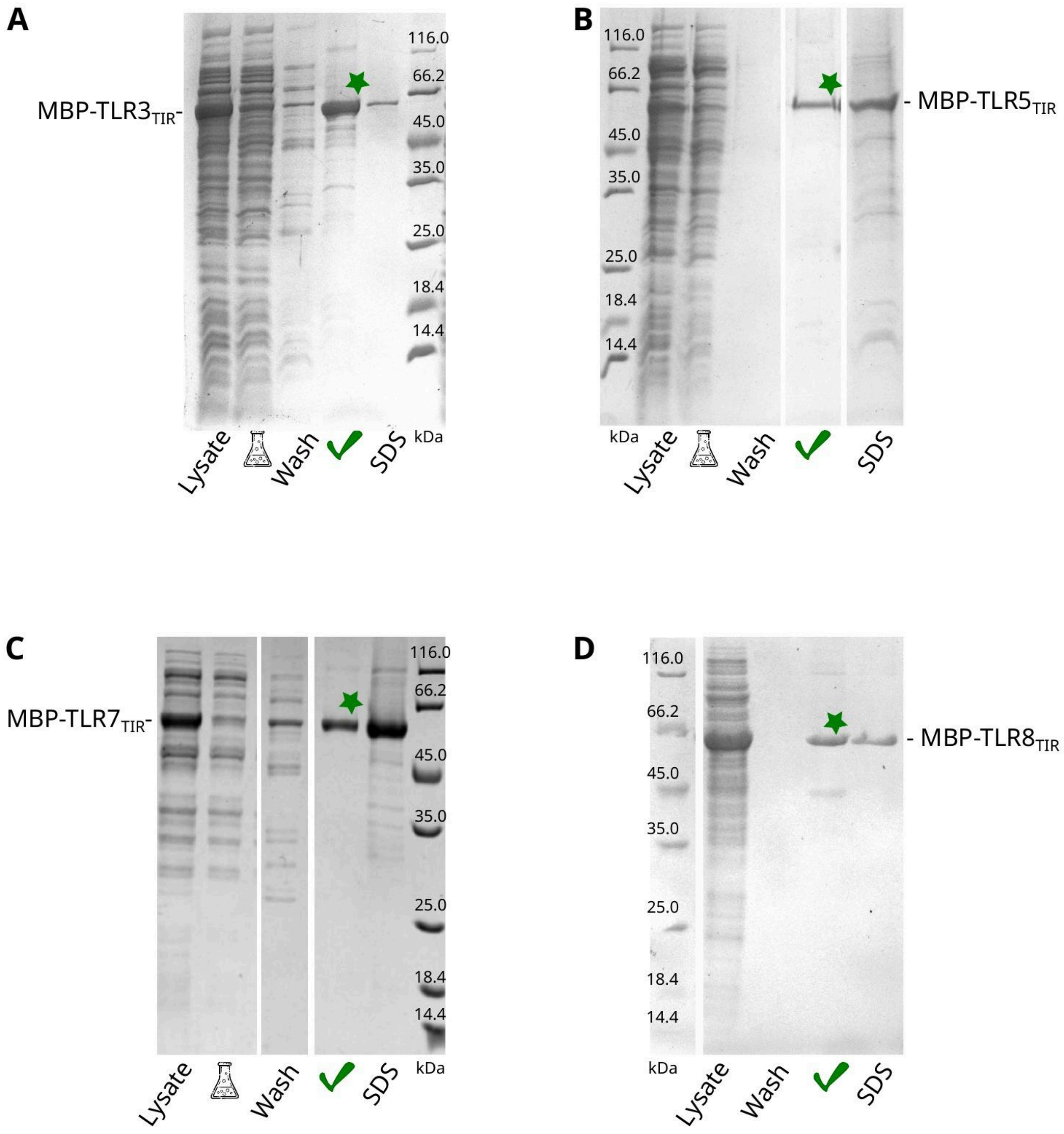

Figure S8. Purification of MBP-tagged TLR3$_{TIR}$ (A), TLR5$_{TIR}$ (B), TLR7$_{TIR}$ (C), TLR8$_{TIR}$ (D) by immobilized metal affinity chromatography (IMAC). *SDS-page lines corresponding to the main fractions of the IMAC are presented. Lines from different gels are separated with white space. "Lysate" - clarified lysate applied to the column; picture of a flask - IMAC flowthrough; "wash" – low imidazole eluate; green mark - purified MBP-TLR$_{TIR}$ fraction; "SDS" - fraction of the protein precipitated on column during purification step (loaded not in equivalent to purified protein fraction). The green star denotes purified MBP-TLR$_{TIR}$ band. Molecular weights marker is loaded and protein bands are signed.*

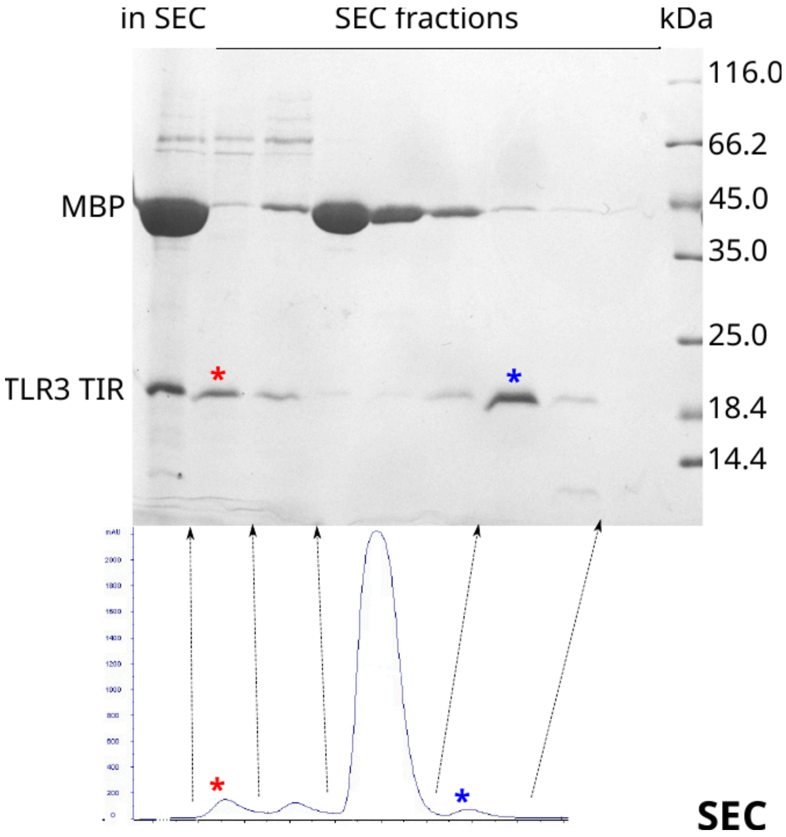

Figure S9. Purification of TLR3$_{TIR}$ after thrombin cleavage from MBP-moiety by size-exclusion chromatography (SEC). *From the top: SDS-page for the main fractions of the SEC. "In SEC" - soluble fraction after hybrid cleavage applied to the column; "SEC fractions" - aliquots of main SEC fractions are loaded to the lines. From the bottom: typical chromatogram of the SEC of TLR3$_{TIR}$. Soluble TLR3$_{TIR}$ in aggregated form is marked with red star, in monomer - with blue star. The same fractions at the SDS-page and at the chromatogram are indicated with arrows. Molecular weights marker is loaded from the right of the gel and signed.*

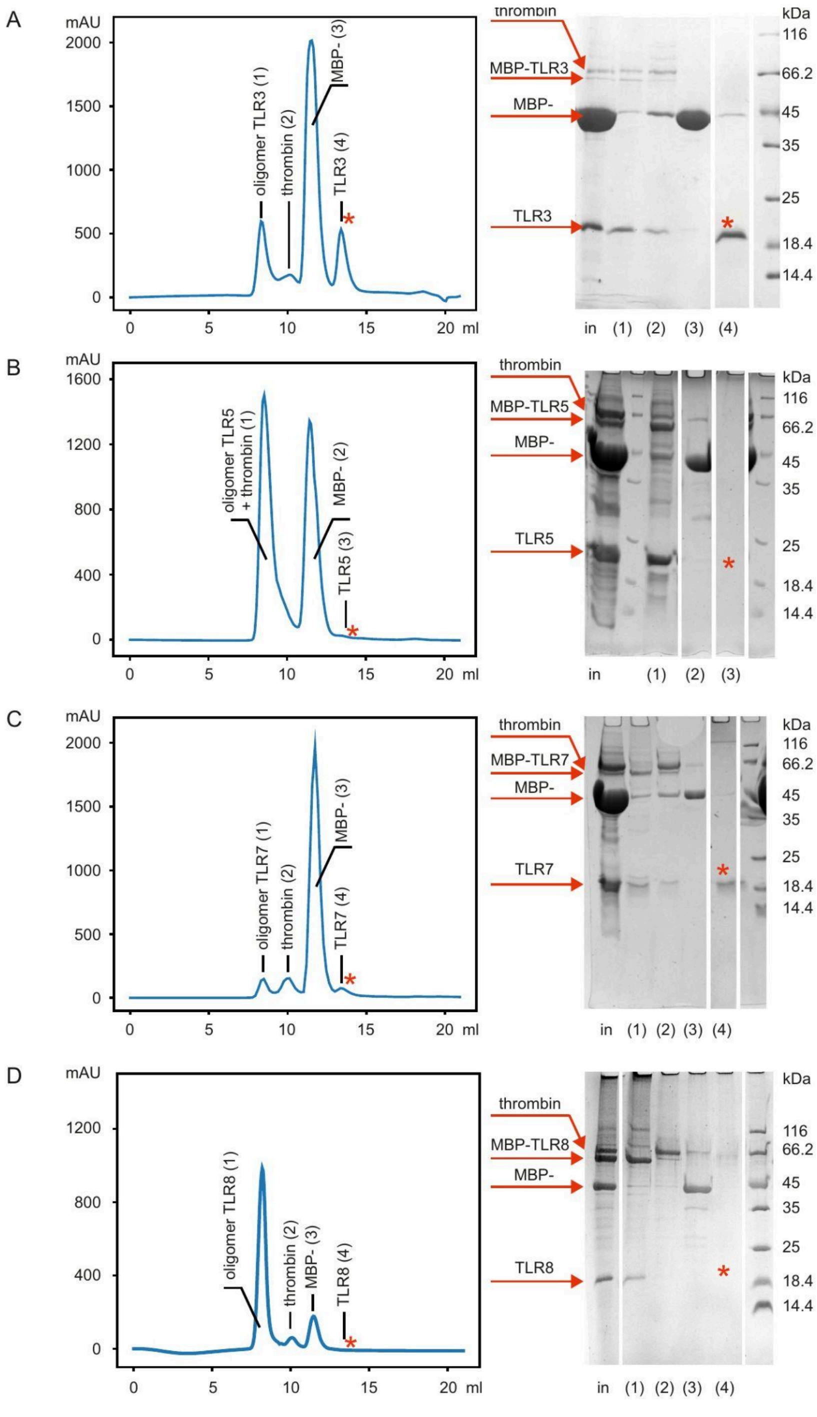

Figure S10. Summary of purification of TLR3/5/7/8$_{TIR}$ after thrombin cleavage from MBP-moiety by size-exclusion chromatography (SEC). *From the left: typical chromatograms for TLR3$_{TIR}$* ***(A)****, TLR5$_{TIR}$* ***(B)****, TLR7$_{TIR}$* ***(C)****, TLR8$_{TIR}$* ***(D)*** *purification with SEC. From the right: SDS-page lines corresponding to the main SEC fractions. Lines from different gels are separated with white spaces. Main protein bands are marked with arrows and signed. Molecular weights marker is loaded from the right of the gel and signed. "In" - soluble fraction after hybrid cleavage applied to the column, "oligomer TLR$_{TIR}$ (1)" - fraction, containing soluble TLR$_{TIR}$ in aggregated state; "thrombin (2)" - fraction containing thrombin; "oligomer TLR$_{TIR}$ + thrombin (1)" - combined fraction containing soluble TLR$_{TIR}$ in aggregated state and thrombin; "MBP (2/3)" - fraction containing MBP-moiety; "TLR$_{TIR}$ (3/4)" - fractions of purified monomeric TLR$_{TIR}$. The red star denotes TLR$_{TIR}$ in monomeric state.*

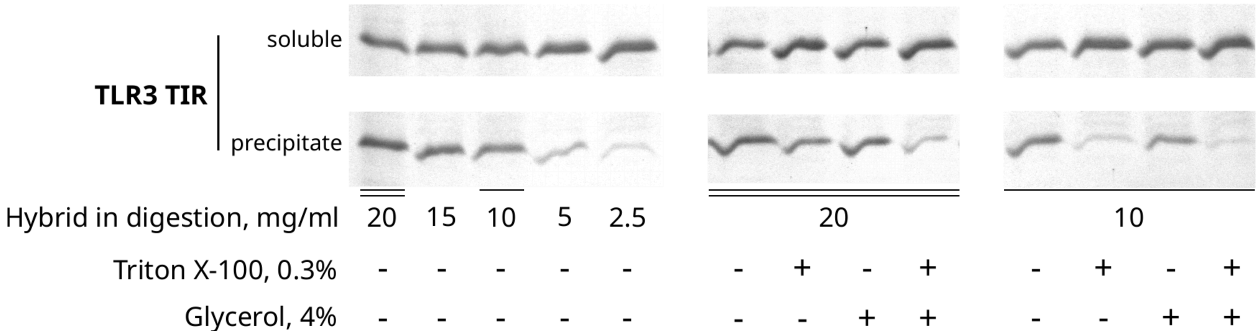

Figure S11. Effectiveness of MBP-TLR$_{TIR}$ digestion depending on hybrid protein concentration and buffer composition. *TLR3$_{TIR}$ was taken as an example. Digestion with thrombin was carried out at 4C for 19h in the presence of 500 mM NaCl in MOPS, pH 8.2. Result of digestion of 2 µg of hybrid is indicated as "soluble" (upper line) and insoluble "precipitate" (lower line) fraction of the reaction. Actual concentration of the hybrid in the reaction and composition of reaction mixture (with ("+") or without ("-") 0.3% Triton X-100 and 4% glycerol) are shown below each pair of wells.*

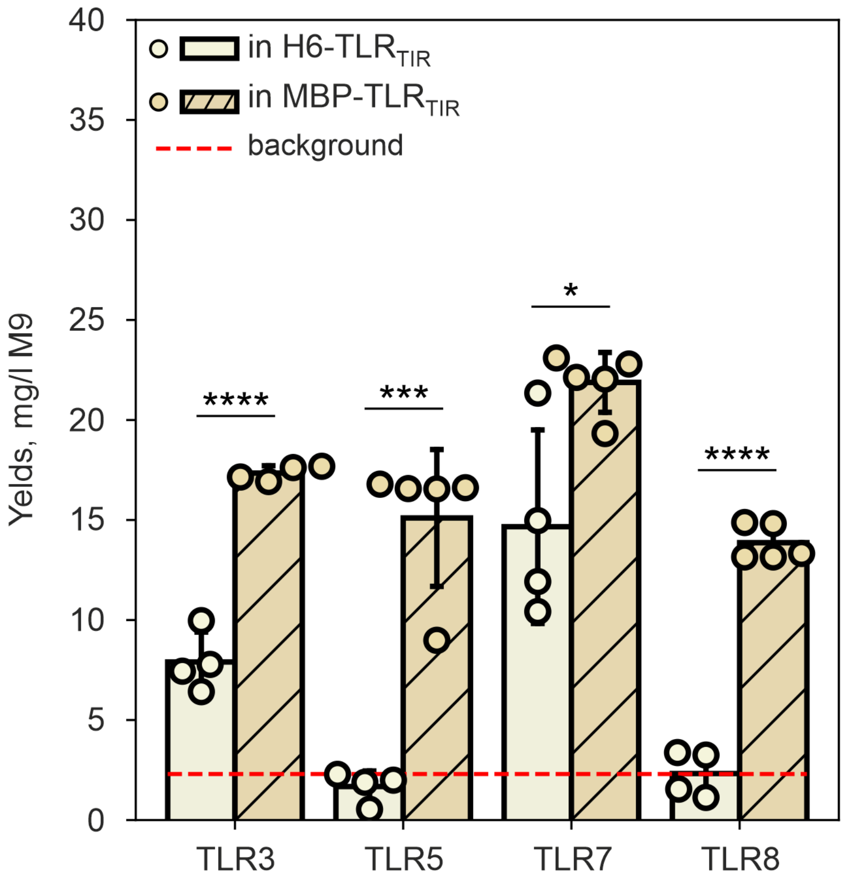

Figure S12. Comparison of yields of the TLR$_{TIR}$-moiety in soluble fraction of H6-TLR$_{TIR}$ and MBP-TLR$_{TIR}$. *Data are presented for TLR3/5/7/8$_{TIR}$ proteins (see also Table S21). In both cases the yield of TLR$_{TIR}$ moiety was derived from actual yield of H6/MBP-TLR$_{TIR}$ hybrid according to their molecular weights. Calculated TLR$_{TIR}$ protein yields are shown in milligrams per liter of M9 minimal salts medium. The yield of the hybrid was quantified based on target protein band intensities on the SDS-PAGE. Statistical significance is provided according to the independed t-test (*-<0.05, **- <0.01, *** - <0.001, **** - <0.0001, ns denotes that the band intensity is not statistically significant compared to the background).*

Table S1. Surface response equations and number of experimental points for H6-TLR1-10$_{TIR}$. *Equations were generated using the linear regression method for constructing contour and surface response plots for H6-TLR1-10$_{TIR}$ yields (Y), depending on the cultivation parameters tested: the temperature after induction (A), time after induction (B), IPTG concentration (C). The number of experimental points used to calculate the surface response equation is indicated in parentheses for each protein.*

Protein (Number of experimental points)	Equation for contour and surface response plots
**H6-TLR1$_{TIR}$ (150 points)**	$Y(TLR1_{TIR}\,mg/l) = 20.985 + 2.055A + 0.889B + 4.953C - 2.423A^2 - - 1.684AB - 4.408AC - 0.849B^2 - 0.339BC - 8.005C^2$
**H6-TLR1$_{TIR}$ (34 points)**	$Y(TLR1_{TIR}\,mg/l) = 17.799 + 7.787A + 0.834B + 1.243C - 4.391A^2 - - 2.108AB - 6.997AC - 1.262B^2 + 0.239BC - 0.171C^2$
**H6-TLR1$_{TIR}$ (27 points)**	$Y(TLR1_{TIR}\,mg/l) = 17.674 + 8.163A - 2.331B - 4.643C - 4.643A^2 - - 1.226AB - 6.443AC - 1.304B^2 + 1.936BC + 3.598C^2$
**H6-TLR1$_{TIR}$ (23 points)**	$Y(TLR1_{TIR}\,mg/l) = 17.844 + 8.849A - 1.710B - 2.092C - 5.119A^2 - - 0.580AB - 6.859AC - 1.438B^2 + 3.663BC + 3.840C^2$
**H6-TLR2$_{TIR}$ (27 points)**	$Y(TLR2_{TIR}\,mg/l) = 18.705 + 12.154A + 3.816B - 6.774C - 7.79A^2 - - 2.665AB - 0.117AC - 2.310B^2 + 1.215BC + 7.489C^2$
**H6-TLR3$_{TIR}$ (23 points)**	$Y(TLR3_{TIR}\,mg/l) = 9.363 + 2.969A + 2.084B - 9.154C - 3.154A^2 - - 1.49AB - 9.154AC - 0.70B^2 - 2.484BC - 25.03C^2$
**H6-TLR4$_{TIR}$ (13 points)**	$Y(TLR4_{TIR}\,mg/l) = 1.61 + 0.439A - 0.012B + 1.996C - - 0.19AB + 0.067AC - 0.025B^2 - 0.186BC + 1.24C^2$
**H6-TLR5$_{TIR}$ (24 points)**	$Y(TLR5_{TIR}\,mg/l) = 0.581 + 0.181A - 0.096B + 0.468C - 0.021A^2 + + 0.049AB + 0.003AC + 0.041B^2 + 0.1BC - 0.093C^2$
**H6-TLR6$_{TIR}$ (35 points)**	$Y(TLR6_{TIR}\,mg/l) = 3.971 + 0.357A - 0.126B - 0.093C - 0.244A^2 - - 0.42AB - 0.137AC - 0.113B^2 + 0.152BC - 0.017C^2$
**H6-TLR7$_{TIR}$ (23 points)**	$Y(TLR7_{TIR}\,mg/l) = 8.256 + 4.949A + 1.906B + 0.755C - 3.330A^2 - - 1.815AB + 0.54AC - 1.885B^2 + 0.089BC - 0.064C^2$
**H6-TLR8$_{TIR}$ (31 points)**	$Y(TLR8_{TIR}\,mg/l) = 1.429 - 0.075A - 0.011B + 0.215C - - 0.215AB + 0.032AC - 0.033B^2 + 0.025BC - 0.05C^2$
**H6-TLR9$_{TIR}$ (24 points)**	$Y(TLR9_{TIR}\,mg/l) = 0.704 + 0.242A + 0.255B - 0.181C - 0.156A^2 - - 0.132AB - 0.019AC - 0.057B^2 + 0.014BC + 0.035C^2$
**H6-TLR10$_{TIR}$ (30 points)**	$Y(TLR10_{TIR}\,mg/l) = 0.554 + 0.183A + 0.263B + 0.066C - 0.06A^2 - - 0.089AB - 0.006AC - 0.076B^2 + 0.021BC - 0.04C^2$

Table S2. Coding scheme for H6-TLR1$_{TIR}$ and H6-TLR2$_{TIR}$ expression parameters. *Coded parameter values are shown for the actual temperature after induction (Temp, ∘C (A)), time after induction (Time, h (B)) and inductor concentration (IPTG, mM (C)).*

	**Parameter value**			
	**-1/-0.25[1]**	**0**	**1**	**2**
**Temp., ℃ (A)**	13	20	28	37
**Time, h (B)**	12	24	36	48
**IPTG, mM (C)**	0	0.01	0.05	-

[1] The value of the first parameter is given for the temperature (A) and time of cultivation of the target proteins (B), the second - for the concentration of IPTG (C).

Table S3. Calculated value of the optimal parameters of H6-TLR1$_{TIR}$ cultivation obtained for different numbers of points used in the regression model. *For surface response equation (Table S1) calculated optima of the function (H6-TLR1$_{TIR}$ yield, mg/l M9 minimal salts medium) and its variables (Temperature after induction, Temp, ∘C; Time after induction, Time, h; and inductor concentration, IPTG, mM) are shown.*

**Number of experimental points**	**150 points**	**34 point**	**27 points**	**23 points**
**Temp., ℃ (A)**	19.80	21.20	23.28	23.52
**Time, h (B)**	29.70	27.50	25.94	25.24
**IPTG, mM (C)**	0.02	0.04	0.04	0.03
**Calculated yield of H6-TLR1$_{TIR}$, mg/l M9**	21.93	19.01	18.56	19.07

Table S4. Coding scheme for Box-Behnken design for H6-TLR1$_{TIR}$. *The table shows the experimental input data for building a model using 150 experimental points. Data used to build a 34-point model are in green. The experimental input data (temperature after induction, Temp, ∘C (A); time after induction, Time, h (B); inductor concentration, IPTG, mM (C)) and the protein yields (tot. - total protein yields, sup. - soluble protein yields in mgs per liter of M9 minimal salts medium), obtained based on the analysis of appropriate band intensity in gel electrophoresis. The coded parameters for the experimental input data are given in columns A, B and C, according to the coding scheme (Table S2).*

**Experimental input data**			**H6-TLR1$_{TIR}$ yield**		**Coded parameters**		
**Temp., ℃ (A)**	**Time, h (B)**	**IPTG,mM (C)**	**Tot., mg/l**	**Sup., mg/l**	**A**	**B**	**C**
20	12	0	11.11	11.06	0.00	-1.00	-0.25
20	24	0	11.68	10.58	0.00	0.00	-0.25
20	36	0	11.49	12.58	0.00	1.00	-0.25
20	12	0.01	13.38	13.59	0.00	-1.00	0.00
20	24	0.01	18.26	16.60	0.00	0.00	0.00
20	36	0.01	17.97	20.27	0.00	1.00	0.00
20	12	0.05	26.66	15.73	0.00	-1.00	1.00
20	24	0.05	69.02	24.49	0.00	0.00	1.00
20	36	0.05	70.70	27.39	0.00	1.00	1.00
28	24	0	24.54	27.54	1.00	0.00	-0.25
28	36	0	23.15	23.41	1.00	1.00	-0.25
28	12	0.01	23.11	17.94	1.00	-1.00	0.00
28	24	0.01	32.21	26.23	1.00	0.00	0.00
28	36	0.01	30.88	20.60	1.00	1.00	0.00
28	24	0.05	46.93	15.85	1.00	0.00	1.00
28	36	0.05	71.15	5.39	1.00	1.00	1.00
37	12	0.00	26.90	21.91	2.00	-1.00	-0.25
37	24	0.00	24.74	20.39	2.00	0.00	-0.25
37	36	0	27.88	13.24	2.00	1.00	-0.25
37	12	0.01	51.80	20.56	2.00	-1.00	0.00
37	24	0.01	45.99	9.91	2.00	0.00	0.00
37	12	0.05	63.33	5.86	2.00	-1.00	1.00
37	24	0.05	60.19	2.32	2.00	0.00	1.00
13	12	0	5.08	4.21	-1.00	-1.00	-0.25
13	24	0	6.70	5.23	-1.00	0.00	-0.25
13	36	0	5.74	4.99	-1.00	1.00	-0.25
13	12	0.01	4.64	4.32	-1.00	-1.00	0.00
13	24	0.01	5.72	5.53	-1.00	0.00	0.00

13	36	0.01	6.27	6.88	-1.00	1.00	0.00
13	12	0.05	9.83	7.67	-1.00	-1.00	1.00
13	24	0.05	16.12	11.53	-1.00	0.00	1.00
13	36	0.05	20.47	12.33	-1.00	1.00	1.00
20	24	0.01	18.26	15.60	0.00	0.00	0.00
20	24	0.01	18.26	17.60	0.00	0.00	0.00
13	48	0.00	5.41	5.03	0.00	2.00	-0.25
13	72	0.00	5.07	4.41	0.00	4.00	-0.25
13	120	0.00	4.48	4.36	0.00	8.00	-0.25
13	0	0.01	2.75	4.30	0.00	-2.00	0.00
13	48	0.01	7.94	7.47	0.00	2.00	0.00
13	72	0.01	8.06	9.14	0.00	4.00	0.00
13	120	0.01	6.60	8.39	0.00	8.00	0.00
13	48	0.05	35.13	14.02	0.00	2.00	1.00
13	72	0.05	35.82	19.10	0.00	4.00	1.00
13	120	0.05	35.43	18.17	0.00	8.00	1.00
13	0	0.05	7.18	6.23	0.00	-2.00	1.00
13	2	0.05	8.17	6.07	0.00	-1.83	1.00
13	4	0.05	9.13	6.28	0.00	-1.67	1.00
13	8	0.05	8.82	6.80	0.00	-1.33	1.00
13	20	0.05	16.73	9.87	0.00	-0.33	1.00
13	28	0.05	25.84	13.27	0.00	0.33	1.00
13	44	0.05	33.47	17.03	0.00	1.67	1.00
13	48	0.05	37.56	37.71	0.00	2.00	1.00
13	52	0.05	40.91	20.29	0.00	2.33	1.00
13	72	0.05	48.16	22.76	0.00	4.00	1.00
13	121	0.05	56.68	24.23	0.00	8.08	1.00
13	144	0.05	66.71	25.45	0.00	10.00	1.00
13	0	0.05	9.51	5.98	0.00	-2.00	1.00
13	2	0.05	7.87	6.40	0.00	-1.83	1.00
13	4	0.05	6.63	6.19	0.00	-1.67	1.00
13	8	0.05	7.28	5.93	0.00	-1.33	1.00
13	20	0.05	11.06	6.17	0.00	-0.33	1.00
13	28	0.05	30.55	21.23	0.00	0.33	1.00
13	44	0.05	38.02	22.77	0.00	1.67	1.00
13	48	0.05	36.39	19.36	0.00	2.00	1.00

13	52	0.05	39.33	20.31	0.00	2.33	1.00
13	72	0.05	46.62	27.01	0.00	4.00	1.00
13	121	0.05	48.63	33.05	0.00	8.08	1.00
13	144	0.05	45.97	36.80	0.00	10.00	1.00
13	0	0.05	8.00	6.22	0.00	-2.00	1.00
13	2	0.05	5.55	4.79	0.00	-1.83	1.00
13	4	0.05	6.63	6.19	0.00	-1.67	1.00
13	8	0.05	7.28	5.93	0.00	-1.33	1.00
13	20	0.05	11.06	6.17	0.00	-0.33	1.00
13	24	0.05	13.28	12.19	0.00	0.00	1.00
13	28	0.05	30.55	21.23	0.00	0.33	1.00
13	44	0.05	38.02	22.77	0.00	1.67	1.00
13	48	0.05	36.39	19.36	0.00	2.00	1.00
13	52	0.05	39.33	20.31	0.00	2.33	1.00
13	72	0.05	46.62	27.01	0.00	4.00	1.00
13	121	0.05	48.63	33.05	0.00	8.08	1.00
13	144	0.05	45.97	36.80	0.00	10.00	1.00
13	0	0.05	8.00	6.22	0.00	-2.00	1.00
13	2	0.05	5.55	4.79	0.00	-1.83	1.00
13	4	0.05	7.24	6.07	0.00	-1.67	1.00
13	8	0.05	9.09	7.50	0.00	-1.38	1.00
13	17	0.05	14.70	9.10	0.00	-0.58	1.00
13	21	0.05	19.83	15.41	0.00	-0.25	1.00
13	24	0.05	23.29	14.22	0.00	0.00	1.00
13	29	0.05	26.77	17.60	0.00	0.42	1.00
13	32	0.05	19.99	12.12	0.00	0.67	1.00
13	48	0.05	29.88	19.18	0.00	2.00	1.00
13	72	0.05	32.70	24.21	0.00	4.00	1.00
13	144	0.05	34.12	28.73	0.00	10.00	1.00
13	0	0.05	7.28	5.75	0.00	-2.00	1.00
13	2	0.05	6.45	5.61	0.00	-1.83	1.00
13	4	0.05	6.78	6.08	0.00	-1.67	1.00
13	8	0.05	7.27	5.53	0.00	-1.38	1.00
13	17	0.05	11.01	8.85	0.00	-0.58	1.00
13	21	0.05	9.35	9.91	0.00	-0.25	1.00
13	29	0.05	15.85	10.79	0.00	0.42	1.00

13	32	0.05	20.94	12.53	0.00	0.67	1.00
13	48	0.05	18.14	13.78	0.00	2.00	1.00
13	72	0.05	19.08	14.15	0.00	4.00	1.00
13	144	0.05	17.40	15.11	0.00	10.00	1.00
13	0	0.05	6.41	5.59	0.00	-2.00	1.00
13	2	0.05	6.14	5.74	0.00	-1.83	1.00
13	4	0.05	6.98	6.02	0.00	-1.67	1.00
13	8	0.05	7.32	5.89	0.00	-1.38	1.00
13	17	0.05	9.36	9.05	0.00	-0.58	1.00
13	21	0.05	11.39	11.93	0.00	-0.25	1.00
13	24	0.05	10.69	8.53	0.00	0.00	1.00
13	29	0.05	10.60	8.86	0.00	0.42	1.00
13	48	0.05	13.30	10.90	0.00	2.00	1.00
13	72	0.05	11.38	12.63	0.00	4.00	1.00
13	144	0.05	14.08	15.60	0.00	10.00	1.00
13	0	1.00	1.88	2.13	0.00	-2.00	24.75
13	48	1.00	43.90	10.69	0.00	2.00	24.75
13	72	1.00	44.93	12.43	0.00	4.00	24.75
13	120	1.00	13.76	11.71	0.00	8.00	24.75
20	0	0.00	12.79	8.70	0.00	-2.00	-0.25
20	2	0.00	12.16	9.10	0.00	-1.83	-0.25
20	8	0.00	11.11	11.06	0.00	-1.33	-0.25
20	20	0.00	11.68	10.58	0.00	-0.33	-0.25
20	44	0.00	10.97	12.69	0.00	1.67	-0.25
20	56	0.00	12.82	13.24	0.00	2.67	-0.25
20	72	0.00	11.29	11.36	0.00	4.00	-0.25
20	92	0.00	10.47	10.84	0.00	5.67	-0.25
20	140	0.00	8.51	12.28	0.00	9.67	-0.25
20	0	0.01	15.25	9.57	0.00	-2.00	0.00
20	2	0.01	13.64	10.86	0.00	-1.83	0.00
20	8	0.01	13.38	13.59	0.00	-1.33	0.00
20	20	0.01	18.26	16.60	0.00	-0.33	0.00
20	44	0.01	18.26	19.34	0.00	1.67	0.00
20	56	0.01	20.24	19.32	0.00	2.67	0.00
20	72	0.01	17.28	16.78	0.00	4.00	0.00
20	92	0.01	15.57	17.80	0.00	5.67	0.00

20	140	0.01	14.41	16.26	0.00	9.67	0.00
20	0	0.05	13.23	10.19	0.00	-2.00	1.00
20	2	0.05	25.02	16.67	0.00	-1.83	1.00
20	44	0.05	72.54	13.03	0.00	1.67	1.00
20	92	0.05	84.17	10.42	0.00	5.67	1.00
20	140	0.05	69.27	2.75	0.00	9.67	1.00
20	1	0.05	5.88	4.56	0.00	-1.92	1.00
20	4	0.05	8.26	7.07	0.00	-1.67	1.00
20	8	0.05	26.66	15.73	0.00	-1.33	1.00
20	48	0.05	67.44	19.38	0.00	2.00	1.00
20	56	0.05	61.60	19.60	0.00	2.67	1.00
20	72	0.05	67.30	23.98	0.00	4.00	1.00
20	120	0.05	60.12	18.66	0.00	8.00	1.00
20	0	0.25	13.18	10.44	0.00	-2.00	6.00
20	2	0.25	15.71	14.02	0.00	-1.83	6.00
20	8	0.25	22.73	16.20	0.00	-1.33	6.00
20	20	0.25	38.43	24.51	0.00	-0.33	6.00
20	44	0.25	49.67	41.87	0.00	1.67	6.00
20	56	0.25	45.70	29.33	0.00	2.67	6.00
20	72	0.25	37.22	27.85	0.00	4.00	6.00
20	92	0.25	36.32	29.52	0.00	5.67	6.00
20	140	0.25	37.24	31.68	0.00	9.67	6.00
20	0	0.40	19.37	16.79	0.00	-2.00	9.75
20	2	0.40	36.46	24.86	0.00	-1.83	9.75
20	8	0.40	72.20	26.78	0.00	-1.33	9.75
20	20	0.40	98.00	22.79	0.00	-0.33	9.75
20	44	0.40	70.57	9.57	0.00	1.67	9.75
20	56	0.40	64.64	8.69	0.00	2.67	9.75
20	72	0.40	62.69	8.21	0.00	4.00	9.75
20	92	0.40	97.13	19.61	0.00	5.67	9.75
20	140	0.40	59.37	2.47	0.00	9.67	9.75
20	0	1.00	16.94	12.02	0.00	-2.00	24.75
20	8	1.00	69.57	33.11	0.00	-1.33	24.75
20	20	1.00	80.13	19.03	0.00	-0.33	24.75
20	92	1.00	51.74	9.56	0.00	5.67	24.75
20	140	1.00	46.83	3.27	0.00	9.67	24.75

28	48	0.00	23.15	23.41	0.00	2.00	-0.25
28	0	0.01	8.14	5.16	0.00	-2.00	0.00
28	4	0.01	9.94	8.29	0.00	-1.67	0.00
28	8	0.01	13.65	11.98	0.00	-1.33	0.00
28	20	0.01	21.58	15.59	0.00	-0.33	0.00
28	28	0.01	24.70	18.66	0.00	0.33	0.00
28	30	0.01	33.67	25.72	0.00	0.50	0.00
28	44	0.01	30.78	21.54	0.00	1.67	0.00
28	48	0.01	31.67	19.89	0.00	2.00	0.00
28	52	0.01	30.94	18.39	0.00	2.33	0.00
28	72	0.01	27.78	15.25	0.00	4.00	0.00
28	121	0.01	30.83	10.62	0.00	8.08	0.00
28	144	0.01	30.04	10.62	0.00	10.00	0.00
28	0	0.01	10.80	8.61	0.00	-2.00	0.00
28	4	0.01	15.15	13.78	0.00	-1.67	0.00
28	8	0.01	18.17	19.97	0.00	-1.33	0.00
28	20	0.01	41.85	28.01	0.00	-0.33	0.00
28	28	0.01	41.06	25.30	0.00	0.33	0.00
28	30	0.01	30.71	18.84	0.00	0.50	0.00
28	44	0.01	28.09	18.63	0.00	1.67	0.00
28	48	0.01	27.95	20.36	0.00	2.00	0.00
28	52	0.01	28.75	22.11	0.00	2.33	0.00
28	72	0.01	31.17	25.39	0.00	4.00	0.00
28	121	0.01	36.82	31.30	0.00	8.08	0.00
28	144	0.01	35.09	42.37	0.00	10.00	0.00
28	0	0.01	9.57	7.08	0.00	-2.00	0.00
28	4	0.01	12.67	9.92	0.00	-1.67	0.00
28	8	0.01	14.11	13.08	0.00	-1.33	0.00
28	20	0.01	29.30	19.02	0.00	-0.33	0.00
28	24	0.01	33.88	22.07	0.00	0.00	0.00
28	28	0.01	33.53	23.33	0.00	0.33	0.00
28	30	0.01	32.74	21.40	0.00	0.50	0.00
28	44	0.01	29.26	17.47	0.00	1.67	0.00
28	48	0.01	27.72	16.99	0.00	2.00	0.00
28	52	0.01	23.60	17.09	0.00	2.33	0.00
28	72	0.01	28.03	20.30	0.00	4.00	0.00

28	121	0.01	31.38	24.06	0.00	8.08	0.00
28	144	0.01	39.40	31.40	0.00	10.00	0.00
28	48	0.05	71.15	5.39	0.00	2.00	1.00
28	48	1.00	62.11	5.84	0.00	2.00	24.75
37	4	0.00	30.46	23.23	0.00	-1.67	-0.25
37	6	0.00	30.47	22.49	0.00	-1.50	-0.25
37	8	0.00	25.32	20.09	0.00	-1.33	-0.25
37	10	0.00	27.06	21.60	0.00	-1.17	-0.25
37	48	0.00	24.13	11.16	0.00	2.00	-0.25
37	0	0.00	8.78	8.07	0.00	-2.00	-0.25
37	2	0.00	18.56	11.94	0.00	-1.83	-0.25
37	4	0.00	19.02	21.04	0.00	-1.67	-0.25
37	8	0.00	43.50	29.58	0.00	-1.33	-0.25
37	10	0.00	58.34	32.08	0.00	-1.17	-0.25
37	20	0.00	55.98	25.11	0.00	-0.33	-0.25
37	28	0.00	29.35	20.84	0.00	0.33	-0.25
37	44	0.00	34.08	17.43	0.00	1.67	-0.25
37	48	0.00	32.72	14.69	0.00	2.00	-0.25
37	52	0.00	31.50	8.95	0.00	2.33	-0.25
37	74	0.00	30.97	5.41	0.00	4.17	-0.25
37	121	0.00	32.18	1.82	0.00	8.08	-0.25
37	0	0.00	8.82	9.53	0.00	-2.00	-0.25
37	2	0.00	22.97	15.83	0.00	-1.83	-0.25
37	4	0.00	38.94	26.59	0.00	-1.67	-0.25
37	8	0.00	77.63	40.51	0.00	-1.33	-0.25
37	10	0.00	58.34	32.08	0.00	-1.17	-0.25
37	20	0.00	55.98	25.11	0.00	-0.33	-0.25
37	24	0.00	41.20	23.77	0.00	0.00	-0.25
37	28	0.00	29.35	20.84	0.00	0.33	-0.25
37	44	0.00	34.08	17.43	0.00	1.67	-0.25
37	48	0.00	32.72	14.69	0.00	2.00	-0.25
37	52	0.00	31.50	8.95	0.00	2.33	-0.25
37	74	0.00	30.97	5.41	0.00	4.17	-0.25
37	121	0.00	32.18	1.82	0.00	8.08	-0.25
37	0	0.00	8.82	9.53	0.00	-2.00	-0.25
37	2	0.00	22.97	15.83	0.00	-1.83	-0.25

37	4	0.00	38.94	26.59	0.00	-1.67	-0.25
37	8	0.00	77.63	40.51	0.00	-1.33	-0.25
37	10	0.00	73.14	39.05	0.00	-1.17	-0.25
37	20	0.00	65.22	19.46	0.00	-0.33	-0.25
37	24	0.00	44.18	15.54	0.00	0.00	-0.25
37	28	0.00	38.18	14.31	0.00	0.33	-0.25
37	44	0.00	36.87	12.73	0.00	1.67	-0.25
37	48	0.00	34.90	11.38	0.00	2.00	-0.25
37	52	0.00	37.62	9.02	0.00	2.33	-0.25
37	74	0.00	31.50	6.00	0.00	4.17	-0.25
37	121	0.00	41.75	1.25	0.00	8.08	-0.25
37	0	0.00	7.72	7.21	0.00	-2.00	-0.25
37	2	0.00	17.53	12.99	0.00	-1.83	-0.25
37	4	0.00	32.35	19.31	0.00	-1.67	-0.25
37	8	0.00	50.39	28.42	0.00	-1.33	-0.25
37	10	0.00	59.35	24.59	0.00	-1.17	-0.25
37	20	0.00	37.61	18.11	0.00	-0.33	-0.25
37	24	0.00	29.75	12.67	0.00	0.00	-0.25
37	28	0.00	27.34	10.82	0.00	0.33	-0.25
37	44	0.00	25.67	9.09	0.00	1.67	-0.25
37	48	0.00	24.72	7.71	0.00	2.00	-0.25
37	52	0.00	24.70	6.59	0.00	2.33	-0.25
37	74	0.00	21.71	5.57	0.00	4.17	-0.25
37	121	0.00	20.85	1.96	0.00	8.08	-0.25
37	0	0.00	4.60	3.42	0.00	-2.00	-0.25
37	1	0.00	11.36	8.31	0.00	-1.92	-0.25
37	2	0.00	22.45	14.60	0.00	-1.83	-0.25
37	4	0.00	23.46	11.75	0.00	-1.67	-0.25
37	6	0.00	34.87	21.01	0.00	-1.50	-0.25
37	9	0.00	39.01	23.90	0.00	-1.25	-0.25
37	19	0.00	49.43	33.65	0.00	-0.42	-0.25
37	0	0.00	4.50	3.72	0.00	-2.00	-0.25
37	1	0.00	13.48	7.05	0.00	-1.92	-0.25
37	2	0.00	22.86	14.39	0.00	-1.83	-0.25
37	4	0.00	27.73	16.98	0.00	-1.67	-0.25
37	6	0.00	33.55	24.33	0.00	-1.50	-0.25

37	9	0.00	37.77	22.36	0.00	-1.25	-0.25
37	19	0.00	42.23	26.05	0.00	-0.42	-0.25
37	0	0.00	17.08	8.90	0.00	-2.00	-0.25
37	1	0.00	13.64	9.72	0.00	-1.92	-0.25
37	2	0.00	28.09	18.52	0.00	-1.83	-0.25
37	4	0.00	32.34	20.32	0.00	-1.67	-0.25
37	6	0.00	45.02	27.16	0.00	-1.50	-0.25
37	9	0.00	44.69	30.58	0.00	-1.25	-0.25
37	19	0.00	55.18	26.81	0.00	-0.42	-0.25
37	4	0.01	59.24	28.42	0.00	-1.67	0.00
37	6	0.01	62.76	27.56	0.00	-1.50	0.00
37	8	0.01	58.80	28.57	0.00	-1.33	0.00
37	10	0.01	52.42	20.96	0.00	-1.17	0.00
37	2	0.05	58.40	21.62	0.00	-1.83	1.00
37	4	0.05	67.36	19.53	0.00	-1.67	1.00
37	6	0.05	57.61	15.69	0.00	-1.50	1.00
37	8	0.05	56.25	14.78	0.00	-1.33	1.00
37	10	0.05	56.41	9.65	0.00	-1.17	1.00
37	2	1.00	50.81	16.33	0.00	-1.83	24.75
37	4	1.00	58.90	18.07	0.00	-1.67	24.75
37	6	1.00	22.52	5.25	0.00	-1.50	24.75
37	8	1.00	72.56	17.83	0.00	-1.33	24.75
37	10	1.00	54.64	11.08	0.00	-1.17	24.75

Table S5. Coding scheme for H6-TLR3-10$_{TIR}$ and MBP-TLR3/5/7/8$_{TIR}$ expression parameters. *Coded parameter values are shown for the actual temperature after induction (Temp, °C (A)), time after induction (Time, h (B)) and inductor concentration (IPTG, mM (C)).*

	Parameter value			
	-1	0	1	2/4[1]
**Temp., °C (A)**	13	20	28	37
**Time, h (B)**	12	24	36	48
**IPTG, mM (C)**	0	0.05	-	0.25

[1] The value of the first parameter is given for the temperature (A) and time of cultivation of the target proteins (B), the second - for the concentration of IPTG (C).

Table S6. Summary on the optimal cultivation parameters and hybrid protein yields for human TLR1-10$_{TIR}$. *Predicted (soluble) and experimental (total in lysate and soluble in clarified lysate) target hybrid protein yields achieved during cellular growth at optimal protein production parameters.*

**Hybrid**		**Optimal cultivation parameters**			**H6/MBP-TLR$_{TIR}$ hybrid yields, mg/l M9**		
**TIR domain**	**N-term tag**	**Temp, ℃**	**IPTG, мM**	**Time, h**	**predicted**	**in lysate**	**in clarified lysate**
					**soluble**	**total**	**soluble**
**TLR1**	His	20	0.01	24	17.8/21.0[1]	42.2±6.8	29.8±3.1
**TLR2**		28	0.01	28	23.2	38.3±1.9	21.5±5.1
**TLR3**		28	0.05	24	8.8	33.0±5.7	8.7±1.7
**TLR4**		28	0.05	24	2.0	39.8±3.2	1.1±0.8
**TLR5**		28	0.25	48	2.0	62.0±5.2	1.8±0.8
**TLR6**		28	0.25	24	2.9	14.7±0.9	4.4±1.4
**TLR7**		28	0.25	30	13.4	49.7±6.1	16.1±5.3
**TLR8**		28	0.25	24	1.5	81.3±9.4	2.5±1.3
**TLR9**		28	0.05	32	0.9	27.9±2.5	0.7±0.5
**TLR10**		28	0.05	32	0.8	54.6±3.9	0.8±0.6
**TLR3**	MBP	13	0.9	70	14.53	67.5±1.1	56.9±1.2
**TLR5**		13	0.25	48	42.9	60.3±2.0	46.9±10.6
**TLR7**		13	0.25	48	61.1	69.9±8.5	69.5±4.8
**TLR8**		28	0.25	24	33.8	51.7±1.8	43.2±2.7

[1] The predicted yields of soluble H6-TLR$_{TIR}$ obtained for models built using 150 / 23 points, respectively.

Table S7. Coding scheme for Box-Behnken design for H6-TLR2$_{TIR}$. *The table shows the experimental input data (temperature after induction, Temp, ℃ (A); time after induction, Time, h (B); inductor concentration, IPTG, mM (C) and protein yields (tot. - total protein yields, sup. - soluble protein yields in mgs per liter of M9 minimal salts medium), obtained based on the analysis of appropriate band intensity in gel electrophoresis. The coded parameters for the experimental input data are given in columns A, B and C, according to the coding scheme (Table S2).*

**Experimental input data**			**H6-TLR2$_{TIR}$ yield**		**Coded parameters**		
**Temp., ℃ (A)**	**Time, h (B)**	**IPTG,mM (C)**	**Tot., mg/l**	**Sup., mg/l**	**A**	**B**	**C**
13	24	0	2.06	0.87	-1.00	0.00	-0.25
13	48	0	0.97	3.90	-1.00	2.00	-0.25
13	24	0.01	2.61	0.95	-1.00	0.00	0.00
13	48	0.01	5.38	2.54	-1.00	2.00	0.00
13	24	0.05	6.74	0.13	-1.00	0.00	1.00
13	48	0.05	14.75	3.08	-1.00	2.00	1.00
28	6	0	22.87	13.79	1.00	-1.50	-0.25
28	24	0	1.61	33.29	1.00	0.00	-0.25
28	48	0	22.87	15.96	1.00	2.00	-0.25
28	6	0.01	15.95	13.42	1.00	-1.50	0.00
28	24	0.01	39.80	29.09	1.00	0.00	0.00
28	48	0.01	27.09	16.08	1.00	2.00	0.00
28	6	0.05	31.70	13.32	1.00	-1.50	1.00
28	24	0.05	34.46	28.92	1.00	0.00	1.00
28	48	0.05	93.41	22.09	1.00	2.00	1.00
37	6	0	28.04	12.84	2.00	-1.50	-0.25
37	24	0	75.39	11.24	2.00	0.00	-0.25
37	48	0	29.42	1.63	2.00	2.00	-0.25
37	6	0.01	32.47	16.48	2.00	-1.50	0.00
37	24	0.01	110.20	8.02	2.00	0.00	0.00
37	48	0.01	32.16	1.47	2.00	2.00	0.00
37	6	0.05	31.54	8.09	2.00	-1.50	1.00
37	24	0.05	68.07	8.09	2.00	0.00	1.00
37	48	0.05	30.64	1.76	2.00	2.00	1.00
28	24	0.01	38.80	20.10	1.00	0.00	0.00
28	24	0.01	38.90	19.10	1.00	0.00	0.00
28	24	0.01	35.60	18.90	1.00	0.00	0.00

Table S8. Coding scheme for Box-Behnken design for H6-TLR3$_{TIR}$. *The table shows the experimental input data (temperature after induction, Temp, °C (A); time after induction, Time, h (B); inductor concentration, IPTG, mM (C) and protein yields (tot. - total protein yields, sup. - soluble protein yields in mgs per liter of M9 minimal salts medium), obtained based on the analysis of appropriate band intensity in gel electrophoresis. The coded parameters for the experimental input data are given in columns A, B and C,according to the coding scheme (Table S5).*

Experimental input data			H6-TLR3$_{TIR}$ yield		Coded parameters		
**Temp., °C (A)**	**Time, h (B)**	**IPTG,mM (C)**	**Tot., mg/l**	**Sup., mg/l**	**A**	**B**	**C**
28	24	0	5.36	3.03	1.00	0.00	-1.00
28	48	0	5.18	4.41	1.00	2.00	-1.00
28	24	0.01	7.40	10.17	1.00	0.00	-0.80
28	48	0.01	21.96	14.23	1.00	2.00	-0.80
28	24	0.05	31.15	9.48	1.00	0.00	0.00
28	48	0.05	58.45	8.74	1.00	2.00	0.00
28	2	0.01	0.83	0.67	1.00	-1.83	-0.80
28	6	0.01	1.58	1.38	1.00	-1.50	-0.80
28	24	0.01	6.43	5.94	1.00	0.00	-0.80
28	30	0.01	6.55	5.50	1.00	0.50	-0.80
28	48	0.01	8.22	7.14	1.00	2.00	-0.80
28	2	0.05	5.76	2.94	1.00	-1.83	0.00
28	6	0.05	37.42	8.57	1.00	-1.50	0.00
28	24	0.05	33.35	9.07	1.00	0.00	0.00
28	30	0.05	34.56	8.09	1.00	0.50	0.00
28	48	0.05	33.53	5.51	1.00	2.00	0.00
28	24	0.05	24.62	8.20	1.00	0.00	0.00
28	24	0.05	34.01	10.98	1.00	0.00	0.00
28	24	0.05	35.87	7.07	1.00	0.00	0.00
13	48	0.05	21.00	7.00	-1.00	2.00	0.00
13	24	0.05	15.00	3.00	-1.00	0.00	0.00
37	8	0.05	36.00	1.50	2.00	-1.33	0.00
37	24	0.05	28.00	0.60	2.00	0.00	0.00

Table S9. Coding scheme for Box-Behnken design for H6-TLR4$_{TIR}$. *The table shows the experimental input data (temperature after induction, Temp, °C (A); time after induction, Time, h (B); inductor concentration, IPTG, mM (C) and protein yields (tot. - total protein yields, sup. - soluble protein yields in mgs per liter of M9 minimal salts medium), obtained based on the analysis of appropriate band intensity in gel electrophoresis. The coded parameters for the experimental input data are given in columns A, B and C, according to the coding scheme (Table S5).*

Experimental input data			H6-TLR4$_{TIR}$ yield		Coded parameters		
**Temp., °C (A)**	**Time, h (B)**	**IPTG,mM (C)**	**Tot., mg/l**	**Sup., mg/l**	**A**	**B**	**C**
28	24	0.05	37.10	2.12	1.00	0.00	0.00
28	24	0.05	43.83	2.53	1.00	0.00	0.00
28	24	0.05	40.94	1.00	1.00	0.00	0.00
28	24	0	3.00	1.04	1.00	0.00	-1.00
28	48	0	1.42	1.30	1.00	2.00	-1.00
28	24	0.01	0.82	1.26	1.00	0.00	-0.80
28	48	0.01	1.09	0.93	1.00	2.00	-0.80
28	24	0.05	37.25	2.68	1.00	0.00	0.00
28	48	0.05	35.45	1.42	1.00	2.00	0.00
13	48	0.05	27.00	1.60	-1.00	2.00	0.00
13	24	0.05	19.00	1.00	-1.00	0.00	0.00
13	48	0.01	16.00	0.81	-1.00	2.00	-0.80
13	24	0.01	12.00	0.60	-1.00	0.00	-0.80

Table S10. Coding scheme for Box-Behnken design for H6-TLR5$_{TIR}$. *The table shows the experimental input data (temperature after induction, Temp, ℃ (A); time after induction, Time, h (B); inductor concentration, IPTG, mM (C) and protein yields (tot. - total protein yields, sup. - soluble protein yields in mgs per liter of M9 minimal salts medium), obtained based on the analysis of appropriate band intensity in gel electrophoresis. The coded parameters for the experimental input data are given in columns A, B and C, according to the coding scheme (Table S5).*

Experimental input data			H6-TLR5$_{TIR}$ yield		Coded parameters		
**Temp., ℃ (A)**	**Time, h (B)**	**IPTG,mM (C)**	**Tot., mg/l**	**Sup., mg/l**	**A**	**B**	**C**
13	24	0	0.02	0.07	-1.00	0.00	-1.00
13	48	0	0.05	0.18	-1.00	2.00	-1.00
13	24	0.05	0.04	0.01	-1.00	0.00	0.00
13	48	0.05	0.05	0.03	-1.00	2.00	0.00
13	24	0.25	13.98	0.91	-1.00	0.00	4.00
13	48	0.25	23.49	0.91	-1.00	2.00	4.00
28	17	0	1.31	0.18	1.00	-0.58	-1.00
28	24	0	1.79	0.07	1.00	0.00	-1.00
28	48	0	1.50	0.10	1.00	2.00	-1.00
28	17	0.05	33.39	0.14	1.00	-0.58	0.00
28	24	0.05	17.75	0.26	1.00	0.00	0.00
28	48	0.05	13.07	0.36	1.00	2.00	0.00
28	17	0.25	27.01	1.65	1.00	-0.58	4.00
28	24	0.25	36.71	1.65	1.00	0.00	4.00
28	48	0.25	66.04	2.50	1.00	2.00	4.00
37	3	0	0.18	0.13	2.00	-1.75	-1.00
37	17	0	0.33	0.22	2.00	-0.58	-1.00
37	24	0	0.49	0.10	2.00	0.00	-1.00
37	3	0.05	33.97	1.92	2.00	-1.75	0.00
37	17	0.05	36.19	1.64	2.00	-0.58	0.00
37	24	0.05	43.01	1.31	2.00	0.00	0.00
37	3	0.25	31.50	0.05	2.00	-1.75	4.00
37	17	0.25	26.34	1.16	2.00	-0.58	4.00
37	24	0.25	28.04	0.43	2.00	0.00	4.00

Table S11. Coding scheme for Box-Behnken design for H6-TLR6$_{TIR}$. *The table shows the experimental input data (temperature after induction, Temp, ℃ (A); time after induction, Time, h (B); inductor concentration, IPTG, mM (C) and protein yields (tot. - total protein yields, sup. - soluble protein yields in mgs per liter of M9 minimal salts medium), obtained based on the analysis of appropriate band intensity in gel electrophoresis. The coded parameters for the experimental input data are given in columns A, B and C, according to the coding scheme (Table S5).*

**Experimental input data**			**H6-TLR6$_{TIR}$ yield**		**Coded parameters**		
**Temp., ℃ (A)**	**Time, h (B)**	**IPTG,mM (C)**	**Tot., mg/l**	**Sup., mg/l**	**A**	**B**	**C**
13	24	0	4.42	3.36	-1.00	0.00	-1.00
13	32	0	4.96	3.84	-1.00	0.67	-1.00
13	56	0	5.32	3.51	-1.00	2.67	-1.00
13	24	0.05	4.98	4.03	-1.00	0.00	0.00
13	32	0.05	4.87	3.78	-1.00	0.67	0.00
13	56	0.05	3.32	3.14	-1.00	2.67	0.00
13	24	0.25	6.70	2.58	-1.00	0.00	4.00
13	32	0.25	9.10	3.36	-1.00	0.67	4.00
13	56	0.25	10.41	4.18	-1.00	2.67	4.00
28	8	0	1.63	2.85	1.00	-1.33	-1.00
28	24	0	2.18	2.34	1.00	0.00	-1.00
28	32	0	1.26	1.99	1.00	0.67	-1.00
28	56	0	0.56	1.87	1.00	2.67	-1.00
28	24	0.05	4.41	4.48	1.00	0.00	0.00
28	32	0.05	10.11	3.68	1.00	0.67	0.00
28	56	0.05	5.37	2.06	1.00	2.67	0.00
28	8	0.25	10.23	2.23	1.00	-1.33	4.00
28	24	0.25	13.60	5.51	1.00	0.00	4.00
28	56	0.25	8.41	1.60	1.00	2.67	4.00
37	4	0	16.96	7.44	2.00	-1.67	-1.00
37	8	0	17.41	7.46	2.00	-1.33	-1.00
37	24	0	14.84	5.30	2.00	0.00	-1.00
37	32	0	13.66	2.63	2.00	0.67	-1.00
37	4	0.05	31.92	5.35	2.00	-1.67	0.00
37	8	0.05	32.08	4.16	2.00	-1.33	0.00
37	24	0.05	22.09	2.42	2.00	0.00	0.00
37	32	0.05	22.63	2.36	2.00	0.67	0.00
37	56	0.05	16.88	1.21	2.00	2.67	0.00
37	4	0.25	1.02	0.17	2.00	-1.67	4.00

37	8	0.25	14.05	2.37	2.00	-1.33	4.00
37	24	0.25	12.70	1.31	2.00	0.00	4.00
37	32	0.25	5.80	1.13	2.00	0.67	4.00
28	24	0.25	15.76	4.06	1.00	0.00	4.00
28	24	0.25	14.66	5.51	1.00	0.00	4.00
28	24	0.25	14.81	2.59	1.00	0.00	4.00

Table S12. Coding scheme for Box-Behnken design for H6-TLR7$_{TIR}$. *The table shows the experimental input data (temperature after induction, Temp, ℃ (A); time after induction, Time, h (B); inductor concentration, IPTG, mM (C) and protein yields (tot. - total protein yields, sup. - soluble protein yields in mgs per liter of M9 minimal salts medium), obtained based on the analysis of appropriate band intensity in gel electrophoresis. The coded parameters for the experimental input data are given in columns A, B and C, according to the coding scheme (Table S5).*

**Experimental input data**			**H6-TLR7$_{TIR}$ yield**		**Coded parameters**		
**Temp., ℃ (A)**	**Time, h (B)**	**IPTG,mM (C)**	**Tot., mg/l**	**Sup., mg/l**	**A**	**B**	**C**
13	30	0	0.19	0.16	-1.00	0.50	-1.00
13	48	0	0.24	0.19	-1.00	2.00	-1.00
13	30	0.05	5.35	0.15	-1.00	0.50	0.00
13	48	0.05	19.74	1.14	-1.00	2.00	0.00
13	30	0.25	1.79	0.22	-1.00	0.50	4.00
13	48	0.25	10.77	0.33	-1.00	2.00	4.00
28	8	0	23.19	5.87	1.00	-1.33	-1.00
28	30	0	41.45	3.78	1.00	0.50	-1.00
28	48	0	44.76	3.90	1.00	2.00	-1.00
28	8	0.05	28.83	9.04	1.00	-1.33	0.00
28	30	0.05	41.77	9.86	1.00	0.50	0.00
28	8	0.25	35.77	13.02	1.00	-1.33	4.00
28	30	0.25	54.44	13.08	1.00	0.50	4.00
28	48	0.25	54.16	1.23	1.00	2.00	4.00
37	8	0	30.94	4.69	2.00	-1.33	-1.00
37	30	0	32.16	0.12	2.00	0.50	-1.00
37	4	0.05	26.33	0.71	2.00	-1.67	0.00
37	8	0.05	40.37	2.46	2.00	-1.33	0.00
37	4	0.25	8.74	5.67	2.00	-1.67	4.00
37	30	0.25	37.04	1.34	2.00	0.50	4.00
28	30	0.25	53.37	23.41	2.00	0.50	4.00
28	30	0.25	41.10	16.42	1.00	0.50	4.00
28	30	0.25	49.88	11.43	1.00	0.50	4.00

Table S13. Coding scheme for Box-Behnken design for H6-TLR8$_{TIR}$. *The table shows the experimental input data (temperature after induction, Temp, ∘C (A); time after induction, Time, h (B); inductor concentration, IPTG, mM (C)) and protein yields (tot. - total protein yields, sup. - soluble protein yields in mgs per liter of M9 minimal salts medium), obtained based on the analysis of appropriate band intensity in gel electrophoresis. The coded parameters for the experimental input data are given in columns A, B and C, according to the coding scheme (Table S5).*

**Experimental input data**			**H6-TLR8$_{TIR}$ yield**		**Coded parameters**		
**Temp., ℃ (A)**	**Time, h (B)**	**IPTG,mM (C)**	**Tot., mg/l**	**Sup., mg/l**	**A**	**B**	**C**
13	8	0	2.26	1.55	-1.00	-1.33	-1.00
13	24	0	3.13	1.69	-1.00	0.00	-1.00
13	32	0	3.76	1.75	-1.00	0.67	-1.00
13	48	0	2.70	1.37	-1.00	2.00	-1.00
13	56	0	2.76	1.14	-1.00	2.67	-1.00
13	8	0.05	31.22	0.32	-1.00	-1.33	0.00
13	24	0.05	42.98	1.52	-1.00	0.00	0.00
13	48	0.05	52.59	2.14	-1.00	2.00	0.00
13	56	0.05	51.44	1.20	-1.00	2.67	0.00
13	8	0.25	19.47	0.67	-1.00	-1.33	4.00
13	24	0.25	35.20	1.33	-1.00	0.00	4.00
13	32	0.25	41.59	1.81	-1.00	0.67	4.00
13	48	0.25	44.62	2.01	-1.00	2.00	4.00
13	56	0.25	50.18	2.32	-1.00	2.67	4.00
28	4	0	6.51	1.04	1.00	-1.67	-1.00
28	8	0	6.22	0.98	1.00	-1.33	-1.00
28	24	0	4.32	0.82	1.00	0.00	-1.00
28	32	0	3.90	0.76	1.00	0.67	-1.00
28	48	0	0.57	0.60	1.00	2.00	-1.00
28	4	0.05	29.87	2.01	1.00	-1.67	0.00
28	8	0.05	52.03	2.03	1.00	-1.33	0.00
28	24	0.05	34.36	0.75	1.00	0.00	0.00
28	32	0.05	51.16	1.43	1.00	0.67	0.00
28	48	0.05	47.59	1.24	1.00	2.00	0.00
28	56	0.05	47.29	0.69	1.00	2.67	0.00
28	4	0.25	69.82	2.01	1.00	-1.67	4.00
28	8	0.25	57.98	2.03	1.00	-1.33	4.00
28	24	0.25	56.29	0.75	1.00	0.00	4.00
28	32	0.25	54.25	1.43	1.00	0.67	4.00

28	48	0.25	55.25	1.24	1.00	2.00	4.00
28	56	0.25	65.06	0.69	1.00	2.67	4.00

Table S14. Coding scheme for Box-Behnken design for H6-TLR9$_{TIR}$. *The table shows the experimental input data (temperature after induction, Temp, ∘C (A); time after induction, Time, h (B); inductor concentration, IPTG, mM (C)) and protein yields (tot. - total protein yields, sup. - soluble protein yields in mgs per liter of M9 minimal salts medium), obtained based on the analysis of appropriate band intensity in gel electrophoresis. The coded parameters for the experimental input data are given in columns A, B and C, according to the coding scheme (Table S5).*

**Experimental input data**			**H6-TLR9$_{TIR}$ yield**		**Coded parameters**		
**Temp., °C (A)**	**Time, h (B)**	**IPTG,mM (C)**	**Tot., mg/l**	**Sup., mg/l**	**A**	**B**	**C**
13	24	0	3.27	0.20	-1.00	0.00	-1.00
13	33	0.05	16.69	1.06	-1.00	0.75	0.00
13	50	0.05	27.44	0.52	-1.00	2.17	0.00
13	50	0.25	19.62	1.07	-1.00	2.17	4.00
28	24	0	12.97	1.28	1.00	0.00	-1.00
28	7	0.05	10.48	0.57	1.00	-1.42	0.00
28	24	0.05	20.20	0.71	1.00	0.00	0.00
28	33	0.05	27.48	1.22	1.00	0.75	0.00
28	7	0.25	30.48	0.14	1.00	-1.42	4.00
28	24	0.25	34.50	0.28	1.00	0.00	4.00
28	33	0.25	27.65	0.23	1.00	0.75	4.00
28	51	0.25	22.47	0.74	1.00	2.25	4.00
37	4	0	8.37	0.55	2.00	-1.67	-1.00
37	7	0	16.09	0.77	2.00	-1.42	-1.00
37	33	0	9.83	0.93	2.00	0.75	-1.00
37	4	0.05	17.50	0.48	2.00	-1.67	0.00
37	7	0.05	30.21	0.32	2.00	-1.42	0.00
37	33	0.05	26.98	0.08	2.00	0.75	0.00
37	4	0.25	15.55	0.31	2.00	-1.67	4.00
37	7	0.25	11.01	0.01	2.00	-1.42	4.00
37	33	0.25	18.06	0.52	2.00	0.75	4.00
28	32	0.05	31.36	1.12	1.00	0.67	0.00
28	32	0.05	27.63	0.92	1.00	0.67	0.00
28	32	0.05	25.29	0.54	1.00	0.67	0.00

Table S15. Coding scheme for Box-Behnken design for H6-TLR10$_{TIR}$. *The table shows the experimental input data (temperature after induction, Temp, ∘C (A); time after induction, Time, h (B); inductor concentration, IPTG, mM (C)) and protein yields (tot. - total protein yields, sup. - soluble protein yields in mgs per liter of M9 minimal salts medium), obtained based on the analysis of appropriate band intensity in gel electrophoresis. The coded parameters for the experimental input data are given in columns A, B and C, according to the coding scheme (Table S5).*

Experimental input data			H6-TLR10$_{TIR}$ yield		Coded parameters		
**Temp., °C (A)**	**Time, h (B)**	**IPTG,mM (C)**	**Tot., mg/l**	**Sup., mg/l**	**A**	**B**	**C**
13	24	0	10.82	0.44	-1.00	0.00	-1.00
13	33	0	10.33	0.65	-1.00	0.75	-1.00
13	50	0	9.37	0.38	-1.00	2.17	-1.00
13	24	0.05	34.05	0.92	-1.00	0.00	0.00
13	50	0.05	53.05	0.61	-1.00	2.17	0.00
13	24	0.25	4.65	0.14	-1.00	0.00	4.00
13	33	0.25	9.21	0.20	-1.00	0.75	4.00
13	50	0.25	29.10	0.77	-1.00	2.17	4.00
28	7	0	16.98	0.23	1.00	-1.42	-1.00
28	24	0	20.40	0.52	1.00	0.00	-1.00
28	33	0	14.99	0.26	1.00	0.75	-1.00
28	51	0	6.09	0.20	1.00	2.25	-1.00
28	7	0.05	39.87	0.41	1.00	-1.42	0.00
28	24	0.05	57.64	1.23	1.00	0.00	0.00
28	33	0.05	44.79	0.65	1.00	0.75	0.00
28	51	0.05	59.11	0.51	1.00	2.25	0.00
28	7	0.25	52.10	0.40	1.00	-1.42	4.00
28	24	0.25	47.68	0.81	1.00	0.00	4.00
28	33	0.25	41.53	0.34	1.00	0.75	4.00
28	51	0.25	40.95	0.44	1.00	2.25	4.00
37	4	0	30.00	0.56	2.00	-1.67	-1.00
37	24	0	42.50	1.31	2.00	0.00	-1.00
37	33	0	33.45	0.51	2.00	0.75	-1.00
37	24	0.05	49.06	0.09	2.00	0.00	0.00
37	7	0.25	40.50	0.13	2.00	-1.42	4.00
37	24	0.25	42.77	0.28	2.00	0.00	4.00
37	33	0.25	66.13	0.10	2.00	0.75	4.00
28	32	0.05	48.87	0.07	2.00	0.67	0.00
28	32	0.05	56.50	1.24	2.00	0.67	0.00

28	32	0.05	55.25	0.60	2.00	0.67	0.00

Table S16. Surface response equations and number of experimental points for MBP-TLR3/5/7/8$_{TIR}$. *Equations were generated using the linear regression methodfor constructing contour and surface response plots for MBP-TLR3/5/7/8$_{TIR}$ yields (Y), depending on the cultivation parameters tested: the temperature after induction (A), time after induction (B), IPTG concentration (C). The number of experimental points used to calculate the surface response equation is indicated in parentheses for each protein.*

Protein (Number of experimental points)	Equation for contour and surface response plots
**MBP-TLR3$_{TIR}$ (18 points)**	$Y(MBP–TLR3_{TIR}\ mg/l) = 12.64 - 1.093A + 0.303B + 5.656C -$ $- 0.717A^2 - 0.937AB - 2.437AC + 0.605B^2 + 1.363BC - 0.825C^2$
**MBP-TLR5$_{TIR}$ (31 points)**	$Y(MBP–TLR5_{TIR}\ mg/l) = 9.218 - 2.246A + 1.047B + 3.285C +$ $+ 1.265A^2 - 0.361AB - 1.222AC - 1.139B^2 + 0.847BC + 0.442C^2$
**MBP-TLR7$_{TIR}$ (38 points)**	$Y(MBP–TLR7_{TIR}\ mg/l) = 17.619 + 2.611A + 4.443B + 11.954C - 0.$[illegible] $- 5.089AB - 3.207AC - 1.452B^2 + 0.229BC - 1.799C^2$
**MBP-TLR8$_{TIR}$ (34 points)**	$Y(MBP–TLR8_{TIR}\ mg/l) = 17.532 + 7.901A - 0.618B + 0.043C - 8.8$[illegible] $- 3.033AB + 0.188AC - 1.201B^2 + 0.422BC + 1.022C^2$

Table S17. Coding scheme for Box-Behnken design for MBP-TLR3$_{TIR}$. *The table shows the experimental input data (temperature after induction, Temp, ∘C (A); time after induction, Time, h (B); inductor concentration, IPTG, mM (C)) and protein yields (tot. - total protein yields, sup. - soluble protein yields in mgs per liter of M9 minimal salts medium), obtained based on the analysis of appropriate band intensity in gel electrophoresis. The coded parameters for the experimental input data are given in columns A, B and C, according to the coding scheme (Table S5).*

**Experimental input data**			**MBP-TLR3$_{TIR}$ yield**		**Coded parameters**		
**Temp., ℃ (A)**	**Time, h (B)**	**IPTG,mM (C)**	**Tot., mg/l**	**Sup., mg/l**	**A**	**B**	**C**
37	24	0.05	45.00	10.00	2	0	0
37	48	0.05	37.00	7.80	2	2	0
37	24	0.01	43.00	9.50	2	0	-0.8
37	48	0	2.00	1.20	2	2	-1
37	24	0	2.50	1.70	2	0	-1
37	48	0.25	36.00	6.90	2	2	4
13	24	0	7.93	6.96	-1	0	-1
13	48	0	6.73	6.67	-1	2	-1
13	24	0.05	14.87	10.94	-1	0	0
13	48	0.05	17.15	15.91	-1	2	0
13	24	0.25	34.00	32.00	-1	0	4
13	48	0.25	52.00	49.00	-1	2	4
28	24	0	6.00	5.00	1	0	-1
28	48	0	10.00	7.00	1	2	-1
13	72	1	66.49	66.74	-1	4	19
13	72	1	68.93	68.02	-1	4	19
13	72	1	57.82	58.04	-1	4	19
13	72	1	56.28	55.54	-1	4	19

Table S18. Coding scheme for Box-Behnken design for MBP-TLR5$_{TIR}$. *The table shows the experimental input data (temperature after induction, Temp, ∘C (A); time after induction, Time, h (B); inductor concentration, IPTG, mM (C)) and protein yields (tot. - total protein yields, sup. - soluble protein yields in mgs per liter of M9 minimal salts medium), obtained based on the analysis of appropriate band intensity in gel electrophoresis. The coded parameters for the experimental input data are given in columns A, B and C, according to the coding scheme (Table S5).*

**Experimental input data**			**MBP-TLR5$_{TIR}$ yield**		**Coded parameters**		
**Temp., ℃ (A)**	**Time, h (B)**	**IPTG,mM (C)**	**Tot., mg/l**	**Sup., mg/l**	**A**	**B**	**C**
13	4	0	14.48	11.67	-1	-1.67	-1
13	24	0	6.89	3.77	-1	0.00	-1
13	48	0	7.34	4.00	-1	2.00	-1
13	56	0	6.77	4.28	-1	2.67	-1
13	24	0.05	10.03	6.76	-1	0.00	0
13	48	0.05	14.04	8.12	-1	2.00	0
13	56	0.05	15.40	8.42	-1	2.67	0
13	24	0.25	44.83	25.23	-1	0.00	4
13	48	0.25	58.35	27.87	-1	2.00	4
13	56	0.25	55.14	22.81	-1	2.67	4
28	9	0.05	8.45	9.93	1	-1.25	0
28	24	0.05	10.94	10.34	1	0.00	0
28	48	0.05	10.75	12.25	1	2.00	0
28	56	0.05	3.83	2.63	1	2.67	0
28	56	0.25	25.79	12.02	1	2.67	4
37	4	0	7.52	8.32	2	-1.67	-1
37	9	0	5.38	6.64	2	-1.25	-1
37	24	0	8.23	6.55	2	0.00	-1
37	4	0.05	23.27	4.73	2	-1.67	0
37	24	0.05	19.78	4.55	2	0.00	0
37	48	0.05	37.06	5.43	2	2.00	0
37	56	0.05	39.72	4.05	2	2.67	0
37	4	0.25	54.90	13.20	2	-1.67	4
37	9	0.25	48.65	9.65	2	-1.25	4
37	24	0.25	44.83	25.23	2	0.00	4
37	48	0.25	58.35	27.87	2	2.00	4
37	56	0.25	55.14	22.81	2	2.67	4
13	48	0.25	59.13	59.36	-1	2.00	4
13	48	0.25	62.86	62.03	-1	2.00	4

13	48	0.25	51.35	51.54	-1	2.00	4
13	48	0.25	52.09	51.41	-1	2.00	4
13	24	1	48.02198	30.42667	-1	0.00	19
13	48	1	57.23034	24.4988	-1	2.00	19
13	56	1	51.65325	25.063	-1	2.67	19
28	9	1	43.329	10.07078	1	-1.25	19
28	24	1	41.84834	6.779698	1	0.00	19
28	48	1	40.09977	5.676348	1	2.00	19
28	56	1	3.994795	3.01301	1	2.67	19
37	4	1	21.15258	8.095744	2	-1.67	19
37	9	1	19.75317	8.409245	2	-1.25	19
37	24	1	35.17297	11.05657	2	0.00	19
37	48	1	39.98519	9.785735	2	2.00	19

Table S19. Coding scheme for Box-Behnken design for MBP-TLR7$_{TIR}$. *The table shows the experimental input data (temperature after induction, Temp, ∘C (A); time after induction, Time, h (B); inductor concentration, IPTG, mM (C)) and protein yields (tot. - total protein yields, sup. - soluble protein yields in mgs per liter of M9 minimal salts medium), obtained based on the analysis of appropriate band intensity in gel electrophoresis. The coded parameters for the experimental input data are given in columns A, B and C, according to the coding scheme (Table S5).*

**Experimental input data**			**MBP-TLR7$_{TIR}$ yield**		**Coded parameters**		
**Temp., ℃ (A)**	**Time, h (B)**	**IPTG,mM (C)**	**Tot., mg/l**	**Sup., mg/l**	**A**	**B**	**C**
13	24	0	7.93	6.96	-1	0.00	-1
13	48	0	6.73	6.67	-1	2.00	-1
13	8	0.05	8.36	7.07	-1	-1.33	0
13	24	0.05	14.87	10.94	-1	0.00	0
13	48	0.05	17.15	15.91	-1	2.00	0
13	56	0.05	15.85	14.06	-1	2.67	0
13	8	0.25	25.46	17.72	-1	-1.33	4
13	24	0.25	48.70	39.97	-1	0.00	4
13	48	0.25	63.06	61.37	-1	2.00	4
13	56	0.25	58.24	55.22	-1	2.67	4
28	8	0	23.53	18.38	1	-1.33	-1
28	24	0	10.06	9.27	1	0.00	-1
28	48	0	6.46	8.79	1	2.00	-1
28	56	0	6.64	6.39	1	2.67	-1
28	8	0.05	11.70	16.09	1	-1.33	0
28	24	0.05	14.90	14.90	1	0.00	0
28	48	0.05	16.33	16.33	1	2.00	0
28	56	0.05	11.22	13.61	1	2.67	0
28	8	0.25	36.06	18.71	1	-1.33	4
28	24	0.25	23.25	16.34	1	0.00	4
28	48	0.25	7.48	7.88	1	2.00	4
28	56	0.25	11.49	7.93	1	2.67	4
37	4	0	8.92	6.24	2	-1.67	-1
37	8	0	4.78	4.72	2	-1.33	-1
37	24	0	4.28	4.80	2	0.00	-1
37	32	0	4.18	3.45	2	0.67	-1
37	4	0.05	36.91	15.86	2	-1.67	0
37	8	0.05	51.98	50.41	2	-1.33	0
37	24	0.05	48.36	23.19	2	0.00	0

37	32	0.05	21.74	11.58	2	0.67	0
37	4	0.25	57.95	26.95	2	-1.67	4
37	8	0.25	20.75	21.31	2	-1.33	4
37	24	0.25	15.83	13.79	2	0.00	4
37	32	0.25	25.51	11.32	2	0.67	4
13	48	0.25	63.91	64.15	-1	2.00	4
13	48	0.25	79.68	78.63	-1	2.00	4
13	48	0.25	70.03	70.29	-1	2.00	4
13	48	0.25	73.37	72.41	-1	2.00	4
13	8	1	19.95378	13.30252	-1	-1.33	19
13	24	1	31.94122	21.89232	-1	0.00	19
13	48	1	57.69748	34.69944	-1	2.00	19
13	56	1	49.96444	49.2925	-1	2.67	19
28	8	1	25.54024	8.069587	1	-1.33	19
28	24	1	15.44053	13.87072	1	0.00	19
28	48	1	9.098768	7.378814	1	2.00	19
28	56	1	11.06811	8.540981	1	2.67	19
37	4	1	15.57462	43.04894	2	-1.67	19
37	8	1	15.97487	21.54415	2	-1.33	19
37	24	1	8.877563	22.66232	2	0.00	19
37	32	1	6.63468	7.513532	2	0.67	19

Table S20. Coding scheme for Box-Behnken design for MBP-TLR8$_{TIR}$. *The table shows the experimental input data (temperature after induction, Temp, ∘C (A); time after induction, Time, h (B); inductor concentration, IPTG, mM (C)) and protein yields (tot. - total protein yields, sup. - soluble protein yields in mgs per liter of M9 minimal salts medium), obtained based on the analysis of appropriate band intensity in gel electrophoresis. The coded parameters for the experimental input data are given in columns A, B and C, according to the coding scheme (Table S5).*

**Experimental input data**			**MBP-TLR8$_{TIR}$ yield**		**Coded parameters**		
**Temp., ℃ (A)**	**Time, h (B)**	**IPTG,mM (C)**	**Tot., mg/l**	**Sup., mg/l**	**A**	**B**	**C**
13	8	0	0.95	0.74	-1	-1.33	-1
13	24	0	0.48	0.68	-1	0.00	-1
13	48	0	0.85	0.76	-1	2.00	-1
13	56	0	0.35	0.21	-1	2.67	-1
13	8	0.05	1.47	1.63	-1	-1.33	0
13	24	0.05	3.56	3.11	-1	0.00	0
13	48	0.05	5.81	3.25	-1	2.00	0
13	8	0.25	4.21	3.55	-1	-1.33	4
13	24	0.25	11.22	8.71	-1	0.00	4
13	48	0.25	18.10	18.11	-1	2.00	4
13	56	0.25	17.97	19.29	-1	2.67	4
28	24	0	1.39	1.77	1	0.00	-1
28	56	0	1.39	0.87	1	2.67	-1
28	8	0.05	10.30	6.31	1	-1.33	0
28	24	0.05	7.42	1.74	1	0.00	0
28	48	0.05	5.61	1.46	1	2.00	0
28	8	0.25	52.19	41.60	1	-1.33	4
28	24	0.25	49.39	20.33	1	0.00	4
28	48	0.25	41.71	17.86	1	2.00	4
28	56	0.25	44.01	22.04	1	2.67	4
37	4	0	16.98	7.08	2	-1.67	-1
37	8	0	12.27	7.28	2	-1.33	-1
37	24	0	8.93	5.75	2	0.00	-1
37	4	0.05	30.23	11.80	2	-1.67	0
37	8	0.05	35.17	6.77	2	-1.33	0
37	24	0.05	32.07	9.13	2	0.00	0
37	4	0.25	39.59	15.85	2	-1.67	4
37	8	0.25	42.69	11.86	2	-1.33	4
37	24	0.25	25.22	6.93	2	0.00	4

37	32	0.25	22.86	5.52	2	0.67	4
13	8	1	3.73	2.54	-1	-1.33	19
13	24	1	7.68	6.90	-1	0.00	19
13	48	1	13.63	16.38	-1	2.00	19
13	56	1	14.74	16.73	-1	2.67	19
28	8	1	45.67	37.94	1	-1.33	19
28	24	1	47.14	30.30	1	0.00	19
28	48	1	3.11	4.99	1	2.00	19
28	56	1	36.37	17.71	1	2.67	19
37	4	1	46.90	16.59	2	-1.67	19
37	8	1	35.37	13.22	2	-1.33	19
37	24	1	34.39	7.70	2	0.00	19

Table S21. Effectiveness of soluble TLR$_{TIR}$ accumulation depending on hybrid construction used. *Soluble hybrid and target protein yields (predicted, actual and calculated) are presented. "Predicted" is the yield of a soluble hybrid obtained using Box-Behnken design; "Actual" is the yield of the soluble hybrid obtained in the experiment; "Calculated" is the yield of soluble TLR$_{TIR}$-moiety only derived from "actual" yield of the hybrid taking into account their molecular weight ratios (tablenotes *,**; Table S22). Calculated "multiplication" of the soluble TLR$_{TIR}$- moiety yield being expressed as a part of His-tagged or MBP-tagged hybrid compares the effectiveness of hybrid constructs used. Coefficient of multiplication larger than "one" for all TLR$_{TIR}$ tested highlights that soluble MBP-tagged protein is expressed more efficiently (Figure S11).*

TIR domain	N-term tag	Soluble protein yields, mg/l M9			N-term tag	Soluble protein yields, mg/l M9			Multiplication of TLR$_{TIR}$-moiety yield***
		Hybrid		TLR$_{TIR}$-moiety		Hybrid		TLR$_{TIR}$-moiety	
		predicted	actual	calculated*		predicted	actual	calculated**	
**TLR3**	His	8.8	8.7±1.7	**7.9±1.5**	MBP	14.53	56.9±1.2	**17.3±0.4**	**x2.2**
**TLR5**		2.0	1.8±0.8	**1.7±0.8**		42.9	46.9±10.6	**15.1±3.4**	**x8.9**
**TLR7**		13.4	16.1±5.3	**14.7±4.8**		61.1	69.5±4.8	**21.9±1.5**	**x1.5**
**TLR8**		1.5	2.5±1.3	**2.3±1.2**		33.8	43.2±2.7	**13.9±0.9**	**x6.0**

* The aminoacid portion of TLR$_{TIR}$ unit in the His-tagged construct is about 90-91%

** The aminoacid portion of TLR$_{TIR}$ unit in the MBP-tagged construct is about 29-31%

*** The ratio of the yield of TLR$_{TIR}$ unit being expressed as a part of MBP-tagged protein to its yield being expressed as a part of His-tagged protein.

Table S22. Amino acid sequence of H6/MBP-TLR$_{TIR}$. *Aminoacids extra to the TLR$_{TIR}$ sequences are shown in bold. HHHHHH represents His6 tag, GSGSG - flexible linker, LVPRGS - thrombin recognition site.*

Protein	aa region of TLR	aa sequence of H6/MBP-TLR$_{TIR}$ hybrid
**H6-TLR1$_{TIR}$**	615-786	**MHHHHHHGSGSGLVPRGS**NIPLEELQRNLQFHAFISYSGHDSFWVKNELLPNLEKEGMQICLHERNFVPGKSIVENIITCIEKSYKSIFVLSPNFVQSEWCHYELYFAHHNLFHEGSNSLILILLEPIPQYSIPSSYHKLKSLMARRTYLEWPKEKSKRGLFWANLRAAINIKLTEQAKK
**TLR1$_{TIR}$-H6 [1,2]**	625-786	MLQFHAFISYSGHDSFWVKNELLPNLEKEGMQICLHERNFVPGKSIVENIITCIEKSYKSIFVLSPNFVQSEWCHYELYFAHHNLFHEGSNSLILILLEPIPQYSIPSSYHKLKSLMARRTYLEWPKEKSKRGLFWANLRAAINIKLTEQAKK**LEHHHHHH**
**H6-TLR2$_{TIR}$**	636-784	**MHHHHHHGSGSGLVPRGS**SRKICYDAFVSYSERDAYWVENLMVQELENFNPPFKLCLHKRDFIPGKWIIDNIIDSIEKSHKTVFVLSENFVKSEWCKYELDFSHFRLFDENNDAAILILLEPIEKKAIPQRFCKLRKIMNTKTYLEWPMDEAQREGFWVNLRAAIKS
**TLR2$_{TIR}$ [1,2]**	626-784	MWLQAKRKPRKAPSRNICYDAFVSYSERDAYWVENLMVQELENFNPPFKLCLHKRDFIPGKWIIDNIIDSIEKSHKTVFVLSENFVKSEWCKYELDFSHFRLFDENNDAAILILLEPIEKKAIPQRFCKLRKIMNTKTYLEWPMDEAQREGFWVNLRAAIKS
**H6-TLR3$_{TIR}$**	745-904	**MHHHHHHGSGSGLVPRGS**KEIDRQTEQFEYAAYIIHAYKDKDWVWEHFSSMEKEDQSLKFCLEERDFEAGVFELEAIVNSIKRSRKIIFVITHHLLKDPLCKRFKVHHAVQQAIEQNLDSIILVFLEEIPDYKLNHALCLRRGMFKSHCILNWPVQKERIGAFRHKLQVALGSKNSVH
**H6-TLR4$_{TIR}$**	662-839	**MHHHHHHGSGSGLVPRGS**NIYDAFVIYSSQDEDWVRNELVKNLEEGVPPFQLCLHYRDFIPGVAIAANIIHEGFHKSRKVIVVVSQHFIQSRWCIFEYEIAQTWQFLSSRAGIIFIVLQKVEKTLLRQQVELYRLLSRNTYLEWEDSVLGRHIFWRRLRKALLDGKSWNPEGTVGTGCNWQEATSI
**H6-TLR5$_{TIR}$**	684-858	**MHHHHHHGSGSGLVPRGS**QGTEPDMYKYDAYLCFSSKDFTWVQNALLKHLDTQYSDQNRFNLCFEERDFVPGENRIANIQDAIWNSRKIVCLVSRHFLRDGWCLEAFSYAQGRCLSDLNSALIMVVVGSLSQYQLMKHQSIRGFVQKQQYLRWPEDFQDVGWFLHKLSQQILKKEKEKKKDNNIPLQTVATIS
**H6-TLR6$_{TIR}$**	630-796	**MHHHHHHGSGSGLVPRGS**NIPLEELQRNLQFHAFISYSEHDSAWVKSELVPYLEKEDIQICLHERNFVPGKSIVENIINCIEKSYKSIFVLSPNFVQSEWCHYELYFAHHNLFHEGSNNLILILLEPIPQNSIPNKYHKLKALMTQRTYLQWPKEKSKRGLFWANIRAAFNMKLTLVTENNDVKS
**H6-TLR6$_{TIR}$ [3]**	640-796	LQFHAFISYSEHDSAWVKSELVPYLEKEDIQICLHERNFVPGKSIVENIINCIEKSYKSIFVLSPNFVQSEWCHYELYFAHHNLFHEGSNNLILILLEPIPQNSIPNKYHKLKALMTQRTYLQWPKEKSKRGLFWANIRAAFNMKLTLVTENNDVKS**MKLTLVTENNDVKSLEHHHHHH**
**H6-TLR7$_{TIR}$**	880-1049	**MHHHHHHGSGSGLVPRGS**GYQRLISPDCCYDAFIVYDTKDPAVTEWVLAELVAKLEDPREKHFNLCLEERDWLPGQPVLENLSQSIQLSKKTVFVMTDKYAKTENFKIAFYLSHQRLMDEKVDVIILIFLEKPFQKSKFLQLRKRLCGSSVLEWPTNPQAHPYFWQCLKNALATDNHVAYSQVFKETV

**H6-TLR8$_{TIR}$**	869-1041	**MHHHHHHGSGSGLVPRGS**GYRSLSTSQTFYDAYISYDTKDASVTDWVINELRYH<br>LEESRDKNVLLCLEERDWDPGLAIIDNLMQSINQSKKTVFVLTKKYAKSWNFKT<br>AFYLALQRLMDENMDVIIFILLEPVLQHSQYLRLRQRICKSSILQWPDNPKAEG<br>LFWQTLRNVVLTENDSRYNNMYVDSIKQY
**H6-TLR9$_{TIR}$**	860-1032	**MHHHHHHGSGSGLVPRGS**QSGRDEDALPYDAFVVFDKTQSAVADWVYNELRGQL<br>EECRGRWALRLCLEERDWLPGKTLFENLWASVYGSRKTLFVLAHTDRVSGLLRA<br>SFLLAQQRLLEDRKDVVVLVILSPDGRRSRYVRLRQRLCRQSVLLWPHQPSGQR<br>SFWAQLGMALTRDNHHFYNRNFCQGPTAE
**H6-TLR10$_{TIR}$**	615-811	**MHHHHHHGSGSGLVPRGS**QEQLKRNVRFHAFISYSEHDSLWVKNELIPNLEKED<br>GSILICLYESYFDPGKSISENIVSFIEKSYKSIFVLSPNFVQNEWCHYEFYFAH<br>HNLFHENSDHIILILLEPIPFYCIPTRYHKLKALLEKKAYLEWPKDRRKCGLFW<br>ANLRAAINVNVLATREMYELQTFTELNEESRGSTISLMRTDCL
**H6-TLR10$_{TIR}$ [4]**	622-776	**MHHHHHHSSGVDLGTENLYFQSM**KTTQEQLKRNVRFHAFISYSEHDSLWVKNEL<br>IPNLEKEDGSILICLYESYFDPGKSISENIVSFIEKSYKSIFVLSPNFVQNEWC<br>HYEFYFAHHNLFHENSDHIILILLEPIPFYCIPTRYHKLKALLEKKAYLEWPKD<br>RRKCGLFWANLRAAIN
**MBP-TLR3$_{TIR}$**	745-904	**MKIEEGKLVIWINGDKGYNGLAEVGKKFEKDTGIKVTVEHPDKLEEKFPQVAA<br>TGDGPDIIFWAHDRFGGYAQSGLLAEITPDKAFQDKLYPFTWDAVRYNGKLIA<br>YPIAVEALSLIYNKDLLPNPPKTWEEIPALDKELKAKGKSALMFNLQEPYFTW<br>PLIAADGGYAFKYENGKYDIKDVGVDNAGAKAGLTFLVDLIKNKHMNADTDYS<br>IAEAAFNKGETAMTINGPWAWSNIDTSKVNYGVTVLPTFKGQPSKPFVGVLSA<br>GINAASPNKELAKEFLENYLLTDEGLEAVNKDKPLGAVALKSYEEELAKDPRI<br>AATMENAQKGEIMPNIPQMSAFWYAVRTAVINAASGRQTVDEALKDAQTNSSS<br>NNPWGSGSGHHHHHHGSGSGLVPRGS**KEIDRQTEQFEYAAYIIHAYKDKDWVWE<br>HFSSMEKEDQSLKFCLEERDFEAGVFELEAIVNSIKRSRKIIFVITHHLLKDPL<br>CKRFKVHHAVQQAIEQNLDSIILVFLEEIPDYKLNHALCLRRGMFKSHCILNWP<br>VQKERIGAFRHKLQVALGSKNSVH
**MBP-TLR5$_{TIR}$**	684-858	**MKIEEGKLVIWINGDKGYNGLAEVGKKFEKDTGIKVTVEHPDKLEEKFPQVAA<br>TGDGPDIIFWAHDRFGGYAQSGLLAEITPDKAFQDKLYPFTWDAVRYNGKLIA<br>YPIAVEALSLIYNKDLLPNPPKTWEEIPALDKELKAKGKSALMFNLQEPYFTW<br>PLIAADGGYAFKYENGKYDIKDVGVDNAGAKAGLTFLVDLIKNKHMNADTDYS<br>IAEAAFNKGETAMTINGPWAWSNIDTSKVNYGVTVLPTFKGQPSKPFVGVLSA<br>GINAASPNKELAKEFLENYLLTDEGLEAVNKDKPLGAVALKSYEEELAKDPRI<br>AATMENAQKGEIMPNIPQMSAFWYAVRTAVINAASGRQTVDEALKDAQTNSSS<br>NNPWGSGSGHHHHHHGSGSGLVPRGS**QGTEPDMYKYDAYLCFSSKDFTWVQNAL<br>LKHLDTQYSDQNRFNLCFEERDFVPGENRIANIQDAIWNSRKIVCLVSRHFLRD<br>GWCLEAFSYAQGRCLSDLNSALIMVVVGSLSQYQLMKHQSIRGFVQKQQYLRWP<br>EDFQDVGWFLHKLSQQILKKEKEKKKDNNIPLQTVATIS
**MBP-TLR7$_{TIR}$**	880-1049	**MKIEEGKLVIWINGDKGYNGLAEVGKKFEKDTGIKVTVEHPDKLEEKFPQVAA<br>TGDGPDIIFWAHDRFGGYAQSGLLAEITPDKAFQDKLYPFTWDAVRYNGKLIA<br>YPIAVEALSLIYNKDLLPNPPKTWEEIPALDKELKAKGKSALMFNLQEPYFTW<br>PLIAADGGYAFKYENGKYDIKDVGVDNAGAKAGLTFLVDLIKNKHMNADTDYS<br>IAEAAFNKGETAMTINGPWAWSNIDTSKVNYGVTVLPTFKGQPSKPFVGVLSA<br>GINAASPNKELAKEFLENYLLTDEGLEAVNKDKPLGAVALKSYEEELAKDPRI<br>AATMENAQKGEIMPNIPQMSAFWYAVRTAVINAASGRQTVDEALKDAQTNSSS<br>NNPWGSGSGHHHHHHGSGSGLVPRGS**GYQRLISPDCCYDAFIVYDTKDPAVTEW<br>VLAELVAKLEDPREKHFNLCLEERDWLPGQPVLENLSQSIQLSKKTVFVMTDKY<br>AKTENFKIAFYLSHQRLMDEKVDVIILIFLEKPFQKSKFLQLRKRLCGSSVLEW<br>PTNPQAHPYFWQCLKNALATDNHVAYSQVFKETV

**MBP-TLR8$_{TIR}$**	869-1041	**MKIEEGKLVIWINGDKGYNGLAEVGKKFEKDTGIKVTVEHPDKLEEKFPQVAA<br>TGDGPDIIFWAHDRFGGYAQSGLLAEITPDKAFQDKLYPFTWDAVRYNGKLIA<br>YPIAVEALSLIYNKDLLPNPPKTWEEIPALDKELKAKGKSALMFNLQEPYFTW<br>PLIAADGGYAFKYENGKYDIKDVGVDNAGAKAGLTFLVDLIKNKHMNADTDYS<br>IAEAAFNKGETAMTINGPWAWSNIDTSKVNYGVTVLPTFKGQPSKPFVGVLSA<br>GINAASPNKELAKEFLENYLLTDEGLEAVNKDKPLGAVALKSYEEELAKDPRI<br>AATMENAQKGEIMPNIPQMSAFWYAVRTAVINAASGRQTVDEALKDAQTNSSS<br>NNPWGSGSGHHHHHHGSGSGLVPRGS**GYRSLSTSQTFYDAYISYDTKDASVTDW<br>VINELRYHLEESRDKNVLLCLEERDWDPGLAIIDNLMQSINQSKKTVFVLTKKY<br>AKSWNFKTAFYLALQRLMDENMDVIIFILLEPVLQHSQYLRLRQRICKSSILQW<br>PDNPKAEGLFWQTLRNVVLTENDSRYNNMYVDSIKQY

Table S23. Gene constructs for expression of TLR1-10$_{TIR}$ proteins.

gene	sequence
**TLR1$_{TIR}$**	atgcatcaccatcaccatcacggttctggttctggtctggttccgcgtggatccaatatcccgctggaggagctgca<br>acgtaacctgcaatttcacgctttcatttcgtacagcggtcatgacagcttctgggttaaaaatgaactgctcccaa<br>atctggaaaaagaaggcatgcagatttgtctgcacgaacgcaactttgtgccgggtaaaagcatcgtcgaaaat<br>atcattacgtgcattgaaaagtcgtataaaagcatcttcgtgctgagcccgaactttgtccaaagcgaatggtgtc<br>actatgagctgtacttcgcacatcataacctgttccatgaaggttcgaactcgctgatcctcatcctgctggagccg<br>atccctcaatatagcattccttcgtcgtaccacaaactgaagagcctcatggcacgtcgtacttatctggaatggcc<br>gaaagaaaagagcaagcgcggcctgttttgggcaaacctgcgtgcagccatcaatatcaagctgaccgagca<br>ggcaaaaaag
**TLR2$_{TIR}$**	atgcatcaccatcaccatcacggttctggttctggtctggttccgcgtggatccagccgtaagatttgttacgacgca<br>tttgttagctactcggaacgcgatgcctactgggttgaaaatctgatggttcaggaactggagaacttcaatccgcc<br>ttttaaactgtgcctgcataaacgcgatttcatcccaggcaagtggatcattgacaacattattgacagcatcgaga<br>agtcgcataagaccgtgttcgtcctgtcggagaactttgttaagtcggaatggtgtaagtacgaactggacttcag<br>ccacttccgcctgtttgacgaaaacaatgatgctgcgattctgatcctcctggaaccaatcgaaaaaaaagctatt<br>cctcaacgcttttgcaaactgcgcaaaatcatgaacactaaaacctacctggagtggcctatggatgaggcgca<br>acgcgaaggtttttgggtaaatctgcgtgcagctattaaatcgt
**TLR3$_{TIR}$**	atgcatcaccatcaccatcacggttctggttctggtctggttccgcgtggatccaaagaaatagacagacagaca<br>gaacagtttgaatatgcagcatatataattcatgcctataaagataaggattgggtctgggaacatttctcttcaatg<br>gaaaaggaagaccaatctctcaaattttgtctggaagaaagggactttgaggcgggtgtttttgaactagaagca<br>attgttaacagcatcaaaagaagcagaaaaattatttttgttataacacaccatctattaaaagacccattatgcaa<br>aagattcaaggtacatcatgcagttcaacaagctattgaacaaaatctggattccattatattggttttccttgagga<br>gattccagattataaactgaaccatgcactctgtttgcgaagaggaatgtttaaatctcactgcatcttgaactggcc<br>agttcagaaagaacggataggtgcctttcgtcataaattgcaagtagcacttggatctaaaaactctgtacat
**TLR4$_{TIR}$**	atgcatcaccatcaccatcacggttctggttctggtctggttccgcgtggatccgctggctgcataaagtatggtag<br>aggtgaaaacatctatgatgcctttgttatctactcaagccaggatgaggactgggtaaggaatgagctagtaaa<br>gaatttagaagaaggggtgcctccatttcagctctgccttcactacagagactttattcccggtgtggccattgctgc<br>caacatcatccatgaaggtttccataaaagccgaaaggtgattgttgtggtgtcccagcacttcatccagagccg<br>ctggtgtatctttgaatatgagattgctcagacctggcagtttctgagcagtcgtgctggtatcatcttcattgtcctgca<br>gaaggtggagaagaccctgctcaggcagcaggtggagctgtaccgccttctcagcaggaacacttacctgga<br>gtgggaggacagtgtcctggggcggcacatcttctggagacgactcagaaaagccctgctggatggtaaatca<br>tggaatccagaaggaacagtgggtacaggatgcaattggcaggaagcaacatctatc
**TLR5$_{TIR}$**	atgcatcaccatcaccatcacggttctggttctggtctggttccgcgtggatcccaaggtactgagccggacatgta<br>caagtacgacgcatatctgtgctttagctcgaaagactttacctgggtgcagaatgctctgctgaaacatctcgac<br>actcaatactcggatcagaatcgttttaatctgtgtttcgaggagcgcgatttcgtacctggtgaaaatcgcattgca<br>aacattcaagacgccatctggaatagccgtaagatcgtatgtctggtttcgcgtcattttctgcgcgacggttggtgc<br>ctggaagcctttagctacgctcaaggccgttgcctgagcgacctgaatagcgcgctgattatggtggtagtcggtt<br>cgctgagccagtaccagctgatgaaacatcagtcgattcgtggcttcgtgcaaaagcagcagtatctgcgctgg<br>ccggaggactttcaggacgtgggctggttcctgcacaagctgtcgcagcagattctgaagaaagagaaagaa<br>aaaaaaaaggataacaatatcccgctgcagactgttgccacgatttcgt
**TLR6$_{TIR}$**	atgcatcaccatcaccatcacggttctggttctggtctggttccgcgtggatccaacattcctctggaagagctgca<br>gcgcaatctgcagttccatgcgtttatctcgtattcggagcacgacagcgcttgggttaaatcggaactggtgccgt<br>atctggaaaaagaggacatccaaatctgcctgcatgaacgcaatttcgtcccgggtaaatcgattgtcgagaat<br>atcattaattgcatcgagaaatcgtataagtcgatttttgtactgtcgccgaacttcgtgcagtcggagtggtgtcact<br>acgaactgtacttcgcgcaccataatctgtttcacgagggttcgaataacctgattctcattctgctggaacctattcc<br>gcagaacagcatcccgaataagtatcacaagctgaaggccctgatgactcagcgcacgtatctgcaatggcc<br>aaaagagaaaagcaagcgtggtctgttctgggcgaatatccgcgccgctttcaacatgaaactgaccctggta<br>actgaaaacaacgacgtaaagagct
**TLR7$_{TIR}$**	atgcatcaccatcaccatcacggttctggttctggtctggttccgcgtggatccggttaccaacgcctgatctcgcc

	agattgttgttacgatgctttcattgtttacgataccaaagatccggcagtgaccgagtgggtactggcagaactgg<br>tagccaaactggaggaccctcgtgagaagcattttaatctgtgcctcgaagaacgcgattggctgccgggtcag<br>ccggtactggagaatctctcgcagagcatccaactgtcgaagaaaaccgtgtttgtcatgaccgataaatacgc<br>gaaaactgaaaacttcaagattgcattttatctgtcgcatcaacgcctgatggacgaaaaggttgacgttattatcc<br>tgattttcctggaaaagccgtttcaaaaatcgaagttcctccaactccgtaaacgcctgtgcggcagcagcgtgct<br>ggagtggccgactaatcctcaagcacacccttacttctggcaatgcctgaaaaatgcactggcgaccgacaac<br>catgtggcttactcgcaagtatttaaggagactgtgt
**$TLR8_{TIR}$**	atgcatcaccatcaccatcacggttctggttctggtctggttccgcgtggatccggttatcgctcgctgtcgactagc<br>cagactttctatgatgcctatatctcgtacgataccaaggatgcttcggtcacggactgggtcattaatgaactgcg<br>ctaccatctggaagaatcgcgcgacaagaatgtgctcctgtgtctggaggaacgtgactgggaccctggtctgg<br>ctatcattgacaatctgatgcagtcgatcaatcaaagcaaaaaaactgtattcgtcctgacgaaaaaatacgcg<br>aagtcgtggaattttaaaaccgctttctacctggctctccaacgtctgatggacgaaaacatggatgtgatcatcttc<br>atcctgctggagccagttctgcagcattcgcaatacctgcgccttcgccaacgtatttgcaaaagcagcattctgc<br>agtggccggataacccgaaggctgaaggtctgttttggcaaactctgcgcaatgttgtgctgactgagaacgac<br>agccgctataataatatgtacgtagattcgattaagcagtatt
**$TLR9_{TIR}$**	atgcatcaccatcaccatcacggttctggttctggtctggttccgcgtggatcccagtcgggtcgtgacgaagatgc<br>gctgccgtacgacgccttcgtcgtgttcgacaaaactcagtcggcggtcgcggattgggtctacaacgagctgcg<br>tggtcagctggaggaatgtcgtggccgttgggccctgcgtctttgcctggaagaacgtgactggctgccgggcaa<br>gactctgttcgagaacctgtgggcatcggtgtacggctcgcgcaagactctgttcgtgctggctcacacggatcgt<br>gtctcgggcctgctgcgtgcttcgtttctgctcgctcagcagcgtctgctggaggaccgtaaggacgtggttgtgct<br>ggtcatcctgtcgcctgatggtcgccgtagccgttatgtgcgcctgcgtcagcgtctgtgtcgccagtcggtgctgct<br>gtggccgcaccaaccgagcggtcagcgctcgttctgggctcaactgggcatggctctgactcgcgataatcacc<br>atttttacaaccgtaacttctgtcaaggcccgactgcagagt
**$TLR10_{TIR}$**	atgcatcaccatcaccatcacggttctggttctggtctggttccgcgtggatcccaagagcaactgaagcgtaatg<br>ttcgctttcatgcttttatctcgtatagcgaacacgacagcctgtgggttaagaacgagctgattcctaacctggaaa<br>aagaggacggctcgattctgatttgtctgtatgagtcgtacttcgatccgggtaaatcgatctcggagaacatcgtt<br>agctttattgaaaaatcgtacaagagcattttcgttctgtcgccgaacttcgtacagaatgagtggtgtcattatgagt<br>tttattttgcccaccacaacctgttccacgaaaattcggatcacatcattctgattctcctggaaccgatccctttttatt<br>gtatccctacccgttaccataagctgaaagccctgctcgaaaagaaggcgtacctggagtggccgaaggatcg<br>ccgcaaatgtggcctgttctgggcaaatctgcgtgccgcaatcaatgtcaatgtactggctactcgcgaaatgtat<br>gagctgcagaccttcactgaactgaacgaggagtcgcgcggttcgacgatctcgctgatgcgcactgattgtctg<br>t

Table S24. PCR primers for H6-TLR1-10$_{TIR}$ and MBP-TLR3/5/7/8$_{TIR}$. *Direction of the primers used to obtain TLR$_{TIR}$ proteins (Table S22) is indicated ("forward" or "reverse").*

protein	Primers (5' → 3')	
TLR1$_{TIR}$	forward	ATATAGGATCCAATATCCCGCTGGAG
	reverse	TATCAAGCTTATCACTTTTTTGCC
TLR2$_{TIR}$	forward	ATATAGGATCCAGCCGTAAGATTTGTTACGAC
	reverse	TATCAAGCTTATCACGATTTAATAG
TLR3$_{TIR}$	forward	ATAGGATCCAAAGAAATAGACAGACAGAC
	reverse	ATAAAGCTTACTAATGTACAGAGTTTTTAGA
TLR4$_{TIR}$	forward	ATATACATATGGCTGGCTGCATAAAGTAT
	reverse	ATATAAAGCTTATCAGATAGATGTTGCTTCC
TLR5$_{TIR}$	forward	ATATAGGATCCCAAGGTACTGAGCCG
	reverse	TATCAAGCTTATCAAGAAATCGTG
TLR6$_{TIR}$	forward	ATATAGGATCCAACATTCCTCTGGAAG
	reverse	TATCAAGCTTATCAGCTCTTTACG
TLR7$_{TIR}$	forward	ATATAGGATCCGGTTACCAACGCCTGA
	reverse	TATCAAGCTTATCACACAGTCTC
TLR8$_{TIR}$	forward	ATATAGGATCCGGTTATCGCTCGCTGTC
	reverse	TATCAAGCTTATCAATACTGCTTA
TLR9$_{TIR}$	forward	ATATAGGATCCCAGTCGGGTCGTGACGAAG
	reverse	TATCAAGCTTATCACTCTGCAGTC
TLR10$_{TIR}$	forward	ATATAGGATCCCAAGAGCAACTGAAGCG
	reverse	ATATAGGATCCGGCCAATGTACTCAGACT

Table S25. *Protein properties and pH of lysis buffers for solubility assay. pI values for proteins (see also Table S6) obtained using ExPASy web service [5]****.***

N-terminal tag	Target protein	pI	Lysis buffer pH
H6	$TLR1_{TIR}$	8.94	7.0
	$TLR2_{TIR}$	7.12	8.0
	$TLR3_{TIR}$	8.34	6.8
	$TLR4_{TIR}$	7.84	8.0
	$TLR5_{TIR}$	8.56	7.0
	$TLR6_{TIR}$	7.83	7.0
	$TLR7_{TIR}$	7.14	8.5
	$TLR8_{TIR}$	7.09	8.5
	$TLR9_{TIR}$	9.45	7.5
	$TLR10_{TIR}$	6.98	8.0
MBP	$TLR3_{TIR}$	6.2	8.2
	$TLR5_{TIR}$	6.06	8.2
	$TLR7_{TIR}$	5.87	8.4
	$TLR8_{TIR}$	5.97	6.8

References

1. Tao, X.; Tong, L. Expression, Purification, and Crystallization of Toll/Interleukin-1 Receptor (TIR) Domains. In *Toll-Like Receptors*; McCoy, C.E., O'Neill, L.A.J., Eds.; Methods in Molecular Biology; Humana Press: Totowa, NJ, **2009**; Vol. 517, pp. 81–88 ISBN 978-1-934115-72-5.
2. Xu, Y.; Tao, X.; Shen, B.; Horng, T.; Medzhitov, R.; Manley, J.L.; Tong, L. Structural Basis for Signal Transduction by the Toll/Interleukin-1 Receptor Domains. *Nature* **2000**, *408*, 111–115, doi:10.1038/35040600.
3. Jang, T.; Park, H.H. Crystal Structure of TIR Domain of TLR6 Reveals Novel Dimeric Interface of TIR–TIR Interaction for Toll-Like Receptor Signaling Pathway. *J. Mol. Biol.* **2014**, *426*, 3305–3313, doi:10.1016/j.jmb.2014.07.024.
4. Nyman, T.; Stenmark, P.; Flodin, S.; Johansson, I.; Hammarström, M.; Nordlund, P. The Crystal Structure of the Human Toll-like Receptor 10 Cytoplasmic Domain Reveals a Putative Signaling Dimer. *J. Biol. Chem.* **2008**, *283*, 11861–11865, doi:10.1074/jbc.C800001200.
5. Gasteiger, E. ExPASy: The Proteomics Server for in-Depth Protein Knowledge and Analysis. *Nucleic Acids Res.* **2003**, *31*, 3784–3788, doi:10.1093/nar/gkg563.
