# Supplementary material for: TIR domains of TLR family-from the cell culture to the protein sample for structural studies
Source: PLoS One. 2024 Jul 5;19(7):e0304997. doi: 10.1371/journal.pone.0304997 (PMC11226090; doi:10.1371/journal.pone.0304997)

Figure 2 A

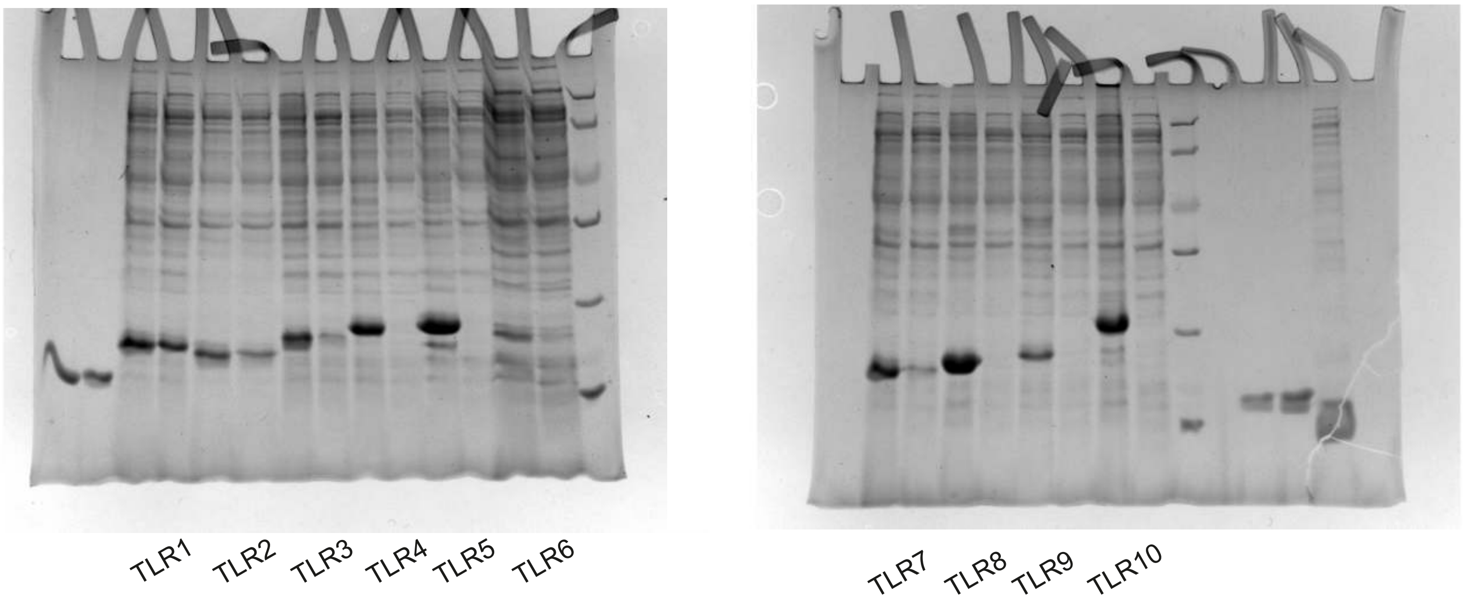

Figure 2 B

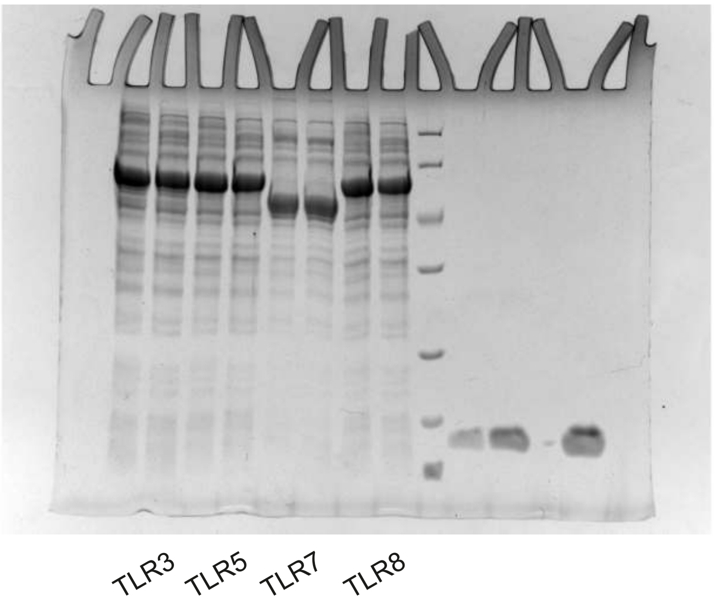

Figure 3 A,B

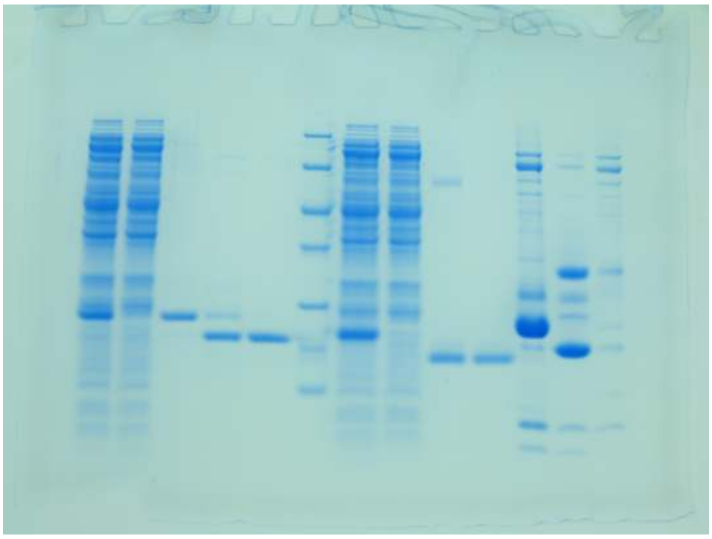

$TLR1_{TIR}$ $TLR2_{TIR}$

Figure 3 C

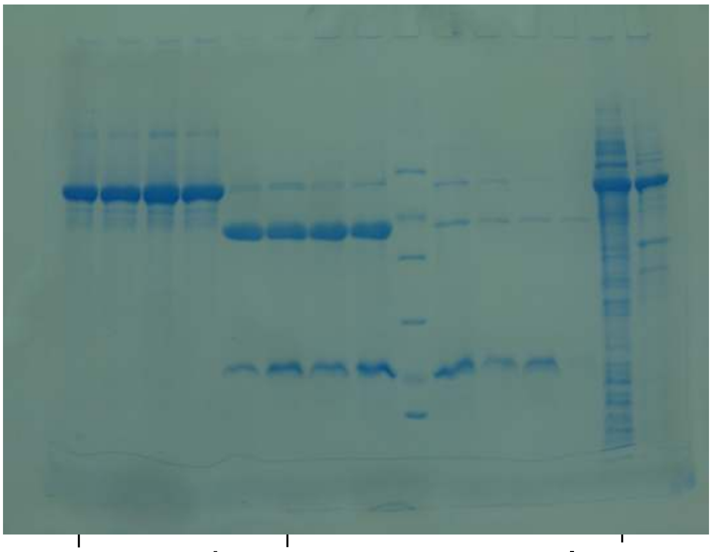

IMAC digestion lysate

Figure 3 C

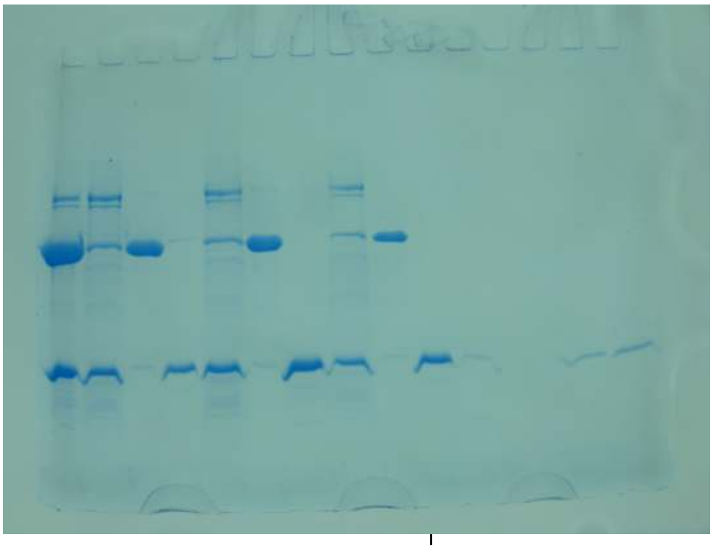

SEC

Figure 3 D

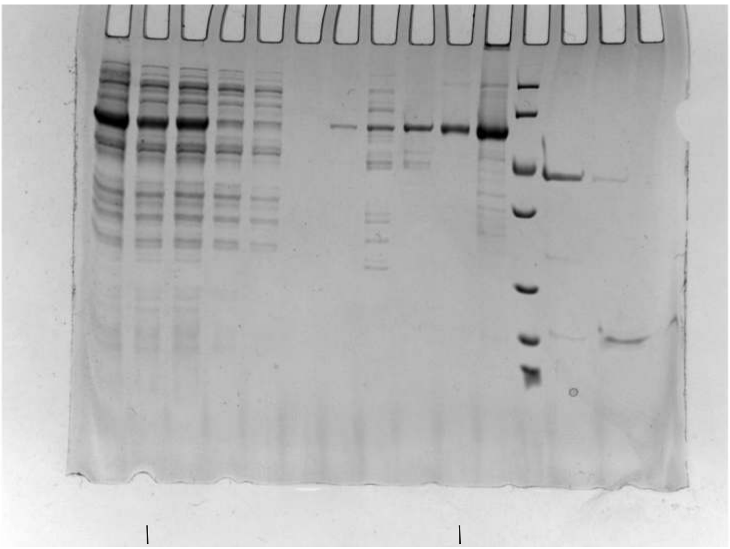

lysate IMAC

Figure 3 D

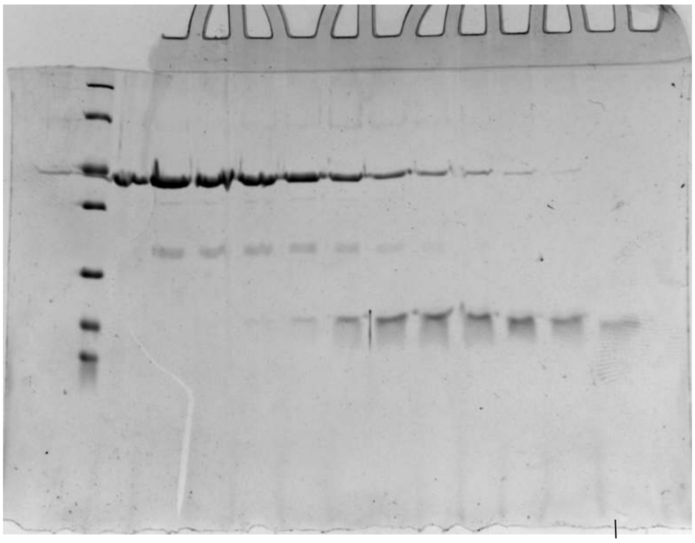

SEC

Figure 3 D

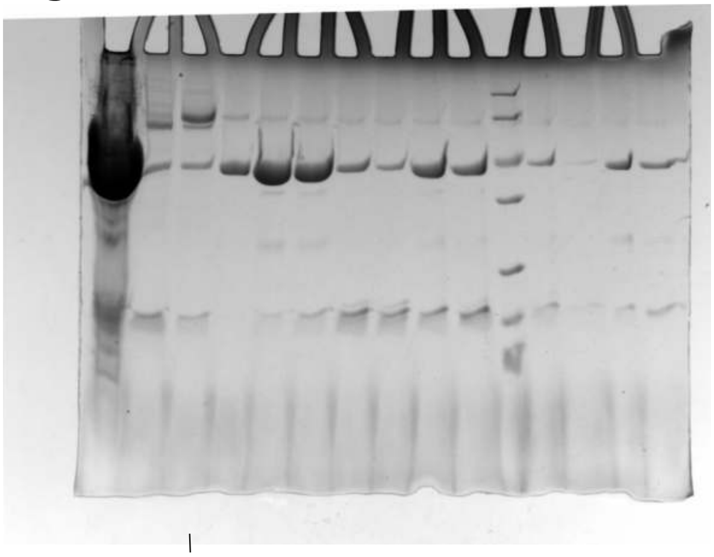

digestion

Figure S7 A

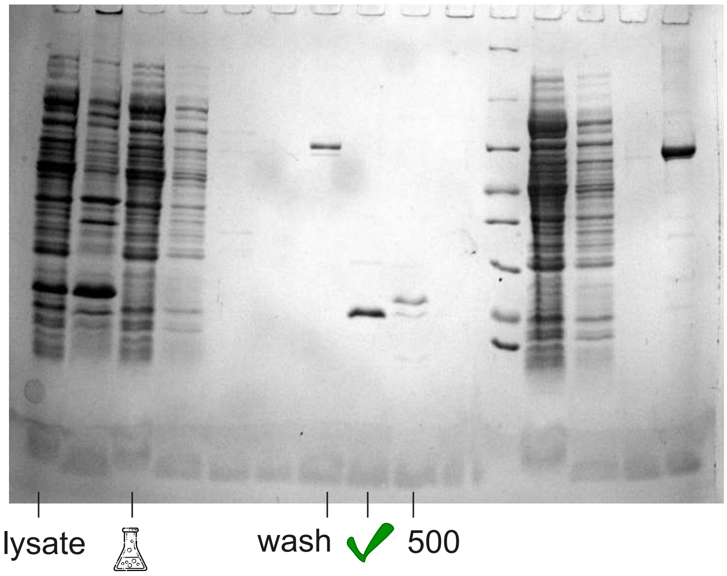

Figure S7 B

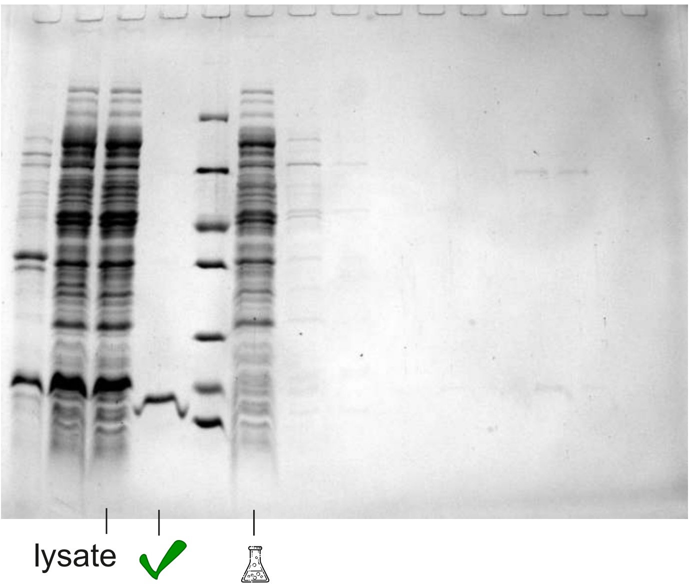

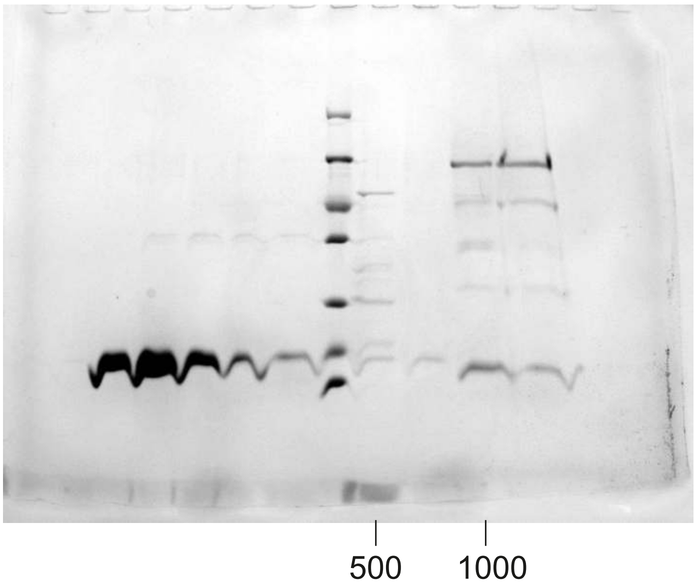

Figure S8 A

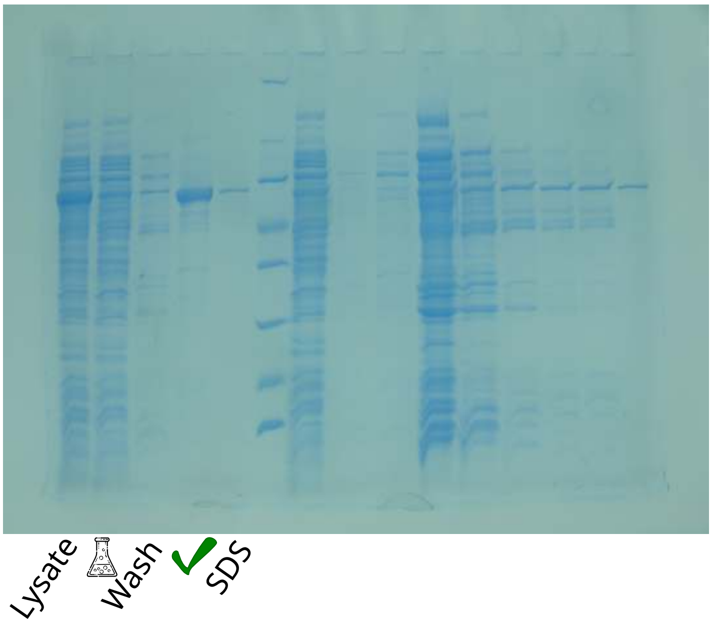

Figure S8 B

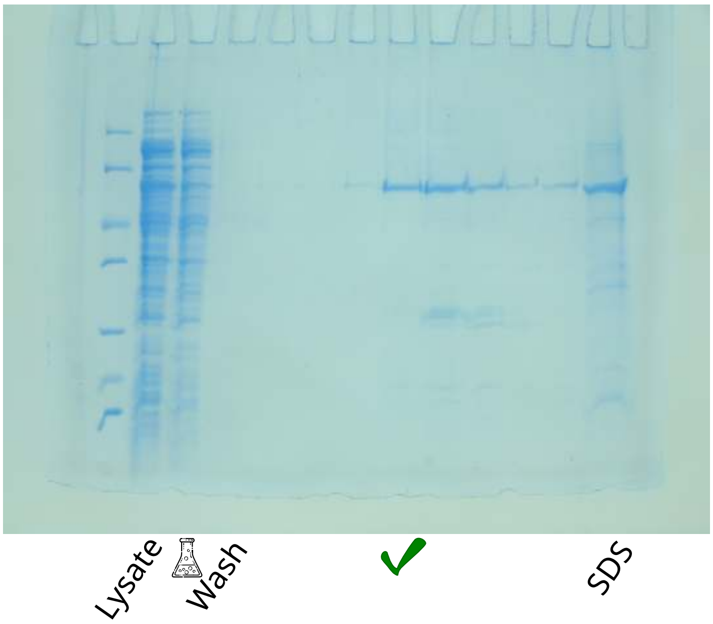

Figure S8 C

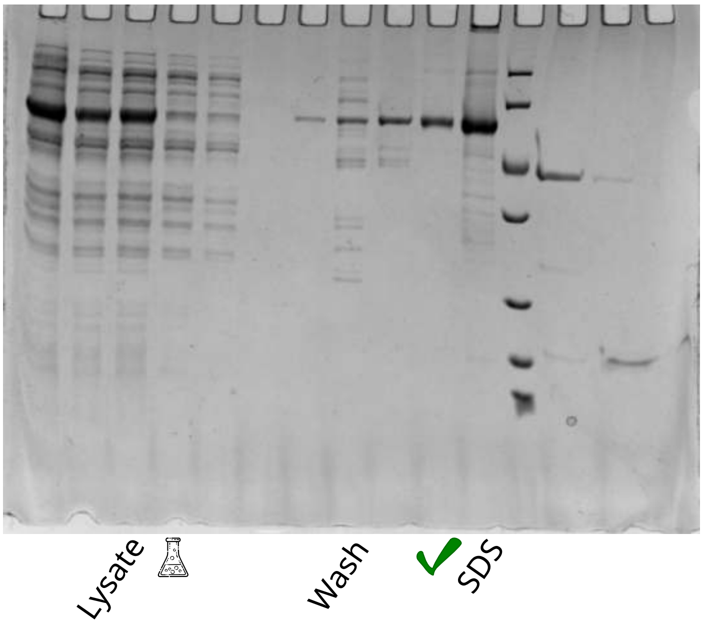

Figure S8 D

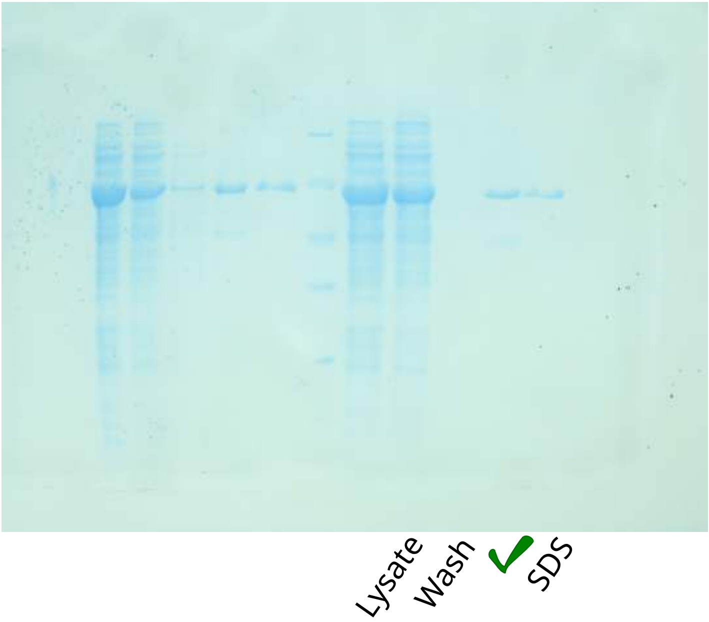

Figure S9

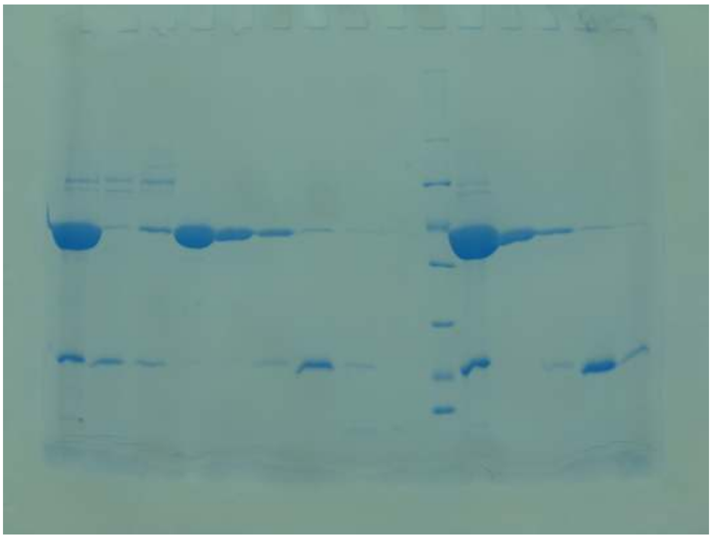

Figure S10A

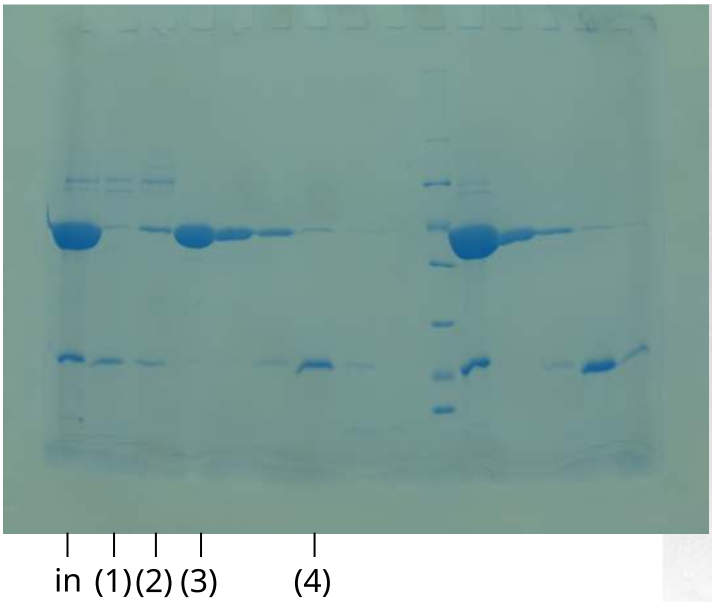

Figure S10B

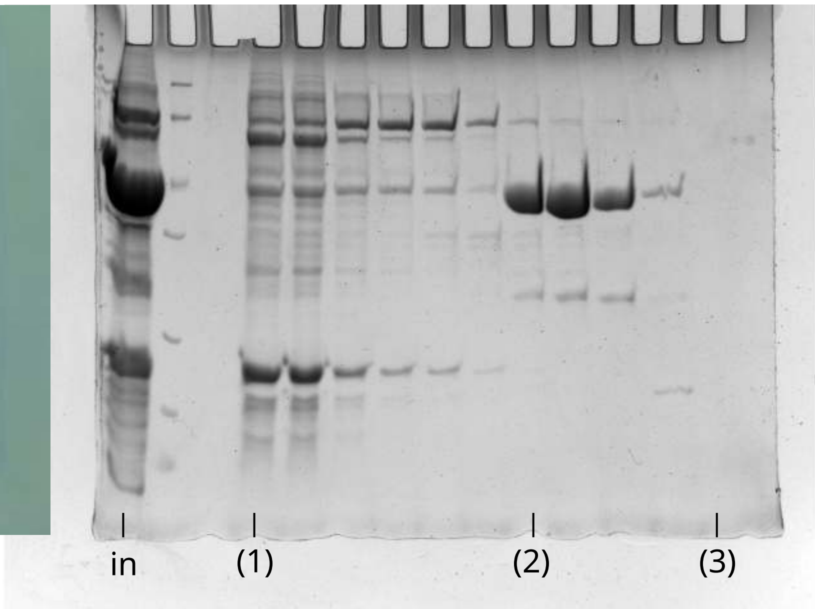

Figure S10C

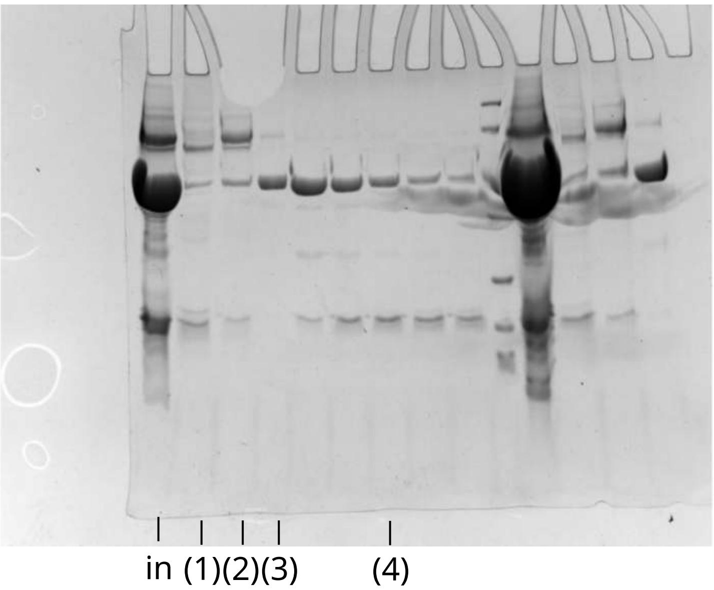

Figure S10D

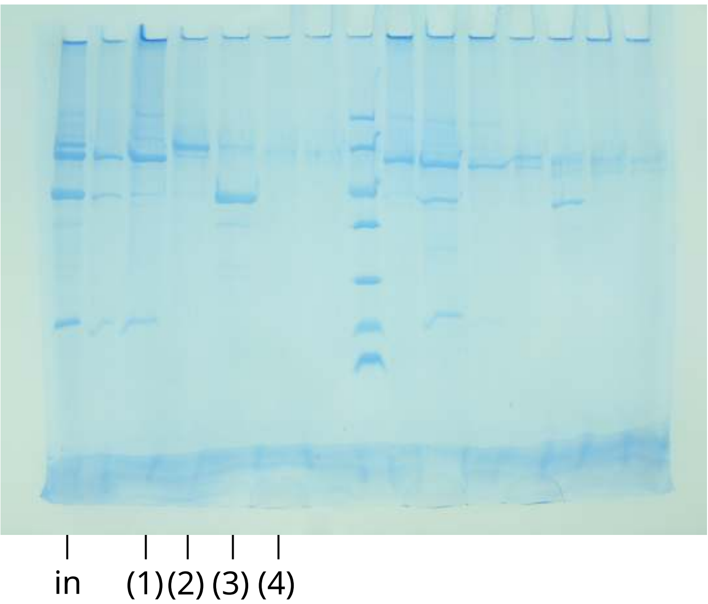

Figure S11

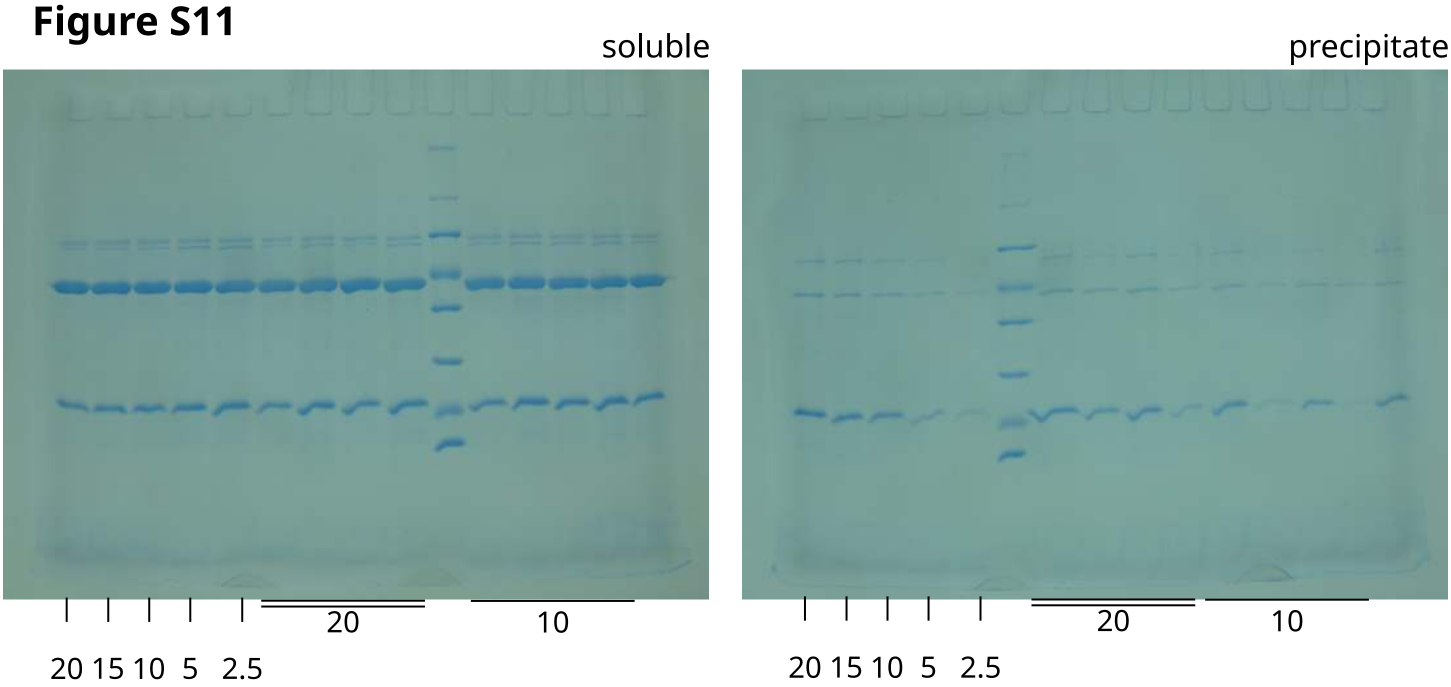

Supplement: S2 File — (PDF) [file pone.0304997.s002.pdf]
