# Supplementary material for: TIR domains of TLR family-from the cell culture to the protein sample for structural studies
Source: PLoS One. 2024 Jul 5;19(7):e0304997. doi: 10.1371/journal.pone.0304997 (PMC11226090; doi:10.1371/journal.pone.0304997)
Supplement: Attachment — Submitted filename: TLR-TIR_Lushpa_etal_Response.pdf [file pone.0304997.s004.pdf]

First of all, we are very grateful to the reviewers for their expertise and opinion. We received several very constructive comments that, as we hope, will improve our manuscript. To answer the comments of the reviewers, we modified the text and figures of the main text (highlighted in cyan) and supplementary materials. Below we provide a point-by-point response to all of the comments.

Reviewer #1: *The manuscript presents interesting data and information; however, a Major Revision is needed as below:*

1. *Please do read and add the following papers to References section of the manuscript to have fruitful Introduction section:*
   Toll-like receptor-guided therapeutic intervention of human cancers: molecular and immunological perspectives. Front Immunol. 2023 Sep 26;14:1244345. doi: 10.3389/fimmu.2023.1244345. PMID: 37822929; PMCID: PMC10562563.
   The Interleukin-1 (IL-1) Superfamily Cytokines and Their Single Nucleotide Polymorphisms (SNPs). J Immunol Res. 2022 Mar 26;2022:2054431. doi: 10.1155/2022/2054431. PMID: 35378905; PMCID: PMC8976653.
   Toll-Like Receptors: General Molecular and Structural Biology. J Immunol Res. 2021 May 29;2021:9914854. doi: 10.1155/2021/9914854. PMID: 34195298; PMCID: PMC8181103.
   The innate and adaptive immune system in human urinary system. Front Immunol. 2023 Oct 9;14:1294869. doi: 10.3389/fimmu.2023.1294869. PMID: 37876936; PMCID: PMC10593411.
   - We added two of the suggested references to the Introduction section that are most closely related to our study: Ref.9 - “Toll-like receptor-guided…” and Ref.5 - “Toll-Like Receptors: General Molecular and Structural Biology”. The other two articles, although related to the immune system, are devoted to other aspects (don’t focus on TLR). The review “The Interleukin-1 (IL-1) Superfamily Cytokines…” focuses on cytokine IL1, not TLR. The editorial article “The innate and adaptive immune system in human urinary system” brings together a series of specialized works in the field of the immunity of the urinary system.

2. *Please do add a flow chart to show all the procedures done within the present study.*
   - We added an additional Figure (now Figure 1) to the main text.

3. *Please do add all the related References for the used protocols.*
   - **We added references to the main text. Moreover, it should be noted that Table 1 includes references to original protocols.**

4. *Please do add the references relating to the applied primers. If the primers were designed in the present study, please do mention the related RefSeq.*
   - We need to note that all genes were designed with codon optimization for the expression in *E. coli*. We included all primer sequences and all DNA sequences of the TLR-TIRs genes used in the work in Supplementary tables S23 and S24. We also presented the final protein constructs in Supplementary table

S22 and now we added the UNIPROT IDs which were used for genes design in the Methods section.

5. *It is recommended to add the structural figures of the related TLRs. PDB is an effective resource in this regard.*

   - **We added the structures of TIR domains of TLR1 (PDB: 7NT7), TLR2 (PDB: 1FYW), and TLR3 (as suggested by AlphaFold) on Figure 4C.**

6. *It is recommended to add statistical analyses to the manuscript to show if there is significant correlation between the related items.*

   - **We added statistical analysis to Figure 3. We also added Figure S12 and modified Table S21.**

7. *It is recommended to interpret the added statistical analyses in Discussion section.*

   - **Based on new Figure S12 we modified the Discussion section, where we compared the efficiency of two types of expression constructs: MBP- and H6-tagged.**

8. *Many of the legends pertaining to the figures and tables are not self-explanatory. Please do revise them.*

   - We modified the legends for Table1 and Figures 2, 3 and 5 in the main text and most of the Figures and Tables in supplementary materials to clarify the data.

9. *Please do revise the Conclusion section.*
10. *It is recommended to add the limitations and the strength of the present study, clearly.*

    - **We modified the Discussion section and now we state: Although our approach is a fast and reliable way to produce TLR_{TIR}, it cannot guarantee the stability of a pure TIR unit. Optimization of expression constructs, such as TIR domain frame or single-point mutations, could expand the palette of available TLR_{TIR} domains for structural studies.**

11. *A mild language polishing is needed.*

    - **We made an additional round of thorough language editing.**

Reviewer #2: *In this manuscript the authors reported experimental methods for the expression and purification of a family of TLR proteins either fused to His-tag of MBP-tag N-terminally. While all experiments are properly carried out and the conclusions are well-supported by the results, I do think all techniques used by the authors are not new as they are routine/classic procedures one would normally consider, so I would say this work fits better in a more specialized journal like Protein Express. Purif. However, I leave it to the Editor to make the final decision as per the guidelines of the Journal, "importance or novelty is not a criteria".*

- **While all methods and techniques are classical, we have described protocols for the production of several TIR domains of TLR in soluble form, as well as their**

purification. Taking into account the lack of structural data on these proteins, we believe that the obtained results will be useful to the scientific community.
